# MSstatsResponse: Semiparametric Statistical Model Enhances Detection of Drug–Protein Interactions in Chemoproteomics Experiments

**DOI:** 10.1016/j.mcpro.2026.101637

**Published:** 2026-08-12

**Authors:** Sarah Szvetecz, Devon Kohler, Joel D. Federspiel, S. Denise Field, Pierre Jean-Beltran, Robert J. Seward, Hyunsuk Suh, Liang Xue, Olga Vitek

**Affiliations:** 1 Khoury College of Computer Science, Northeastern University, Boston, Massachusetts, USA; 2 Barnett Institute for Chemical and Biological Analysis, Northeastern University, Boston, Massachusetts, USA; 3 Preclinical and Translational Sciences, Pfizer Inc., Andover, Massachusetts, USA; 4 Chemical Biology and Proteomics, Pfizer Inc., Cambridge, Massachusetts, USA; 5 Internal Medicine Research Unit, Pfizer Inc., Cambridge, Massachusetts, USA; 6 Machine Learning and Computational Sciences, Pfizer Inc., Cambridge, Massachusetts, USA

**Keywords:** chemoproteomics, quantitative proteomics, mass spectrometry, statistical modeling, dose–response analysis, drug discovery, experimental design

## Abstract

Chemoproteomics is a popular approach for the identification of small molecule–protein interactions in biological systems. Several chemoproteomics workflows leverage functionalized chemical probes and mass spectrometry to measure protein engagement through direct protein enrichment or competition using a range of small molecule concentrations. Statistical methods for analysis of such dose–response chemoproteomics data sets are limited. For example, existing methods rely on fixed curve shapes and are sensitive to experimental variation, particularly when the number of doses or replicates is limited. Here, we present MSstatsResponse, a semiparametric statistical framework for analyzing chemoproteomic dose–response experiments that uses isotonic regression that does not require a fixed curve shape. This approach improves the accuracy and robustness of curve fitting, target identification, and half-response estimation across diverse experimental designs. We evaluate MSstatsResponse by generating a benchmark chemoproteomic data set that profiled the competition between the kinase-binding probe XO44 and the drug Dasatinib using three mass spectrometry acquisition strategies: data-independent acquisition, tandem mass tag–based data-dependent acquisition, and selected reaction monitoring. We further evaluate the method on simulated data sets that vary the number of doses, number of replicates, and levels of noise, and demonstrate that MSstatsResponse consistently improves sensitivity, specificity, and reproducibility compared to existing methods, particularly in low-replicate and low-dose settings. MSstatsResponse is implemented as an open-source R/Bioconductor package that integrates with the MSstats ecosystem for quantitative proteomics. It provides a unified workflow for preprocessing, curve fitting, target identification, and experimental design, enabling researchers to select the number of doses and replicates appropriate to their experimental goals. The software and documentation are freely available at https://bioconductor.org/packages/MSstatsResponse.

## Highlights

• MSstatsResponse fits dose–response curves via isotonic regression.
• Maintains high sensitivity and specificity across diverse experimental designs.
• MSstatsResponse is an open-source R package available on Bioconductor.
• Supports curve fitting, target identification, and IC50 estimation.
• Benchmarked on a XO44–Dasatinib dataset across DIA, TMT, and SRM, plus simulations.

## Graphical Abstract

**Figure:**
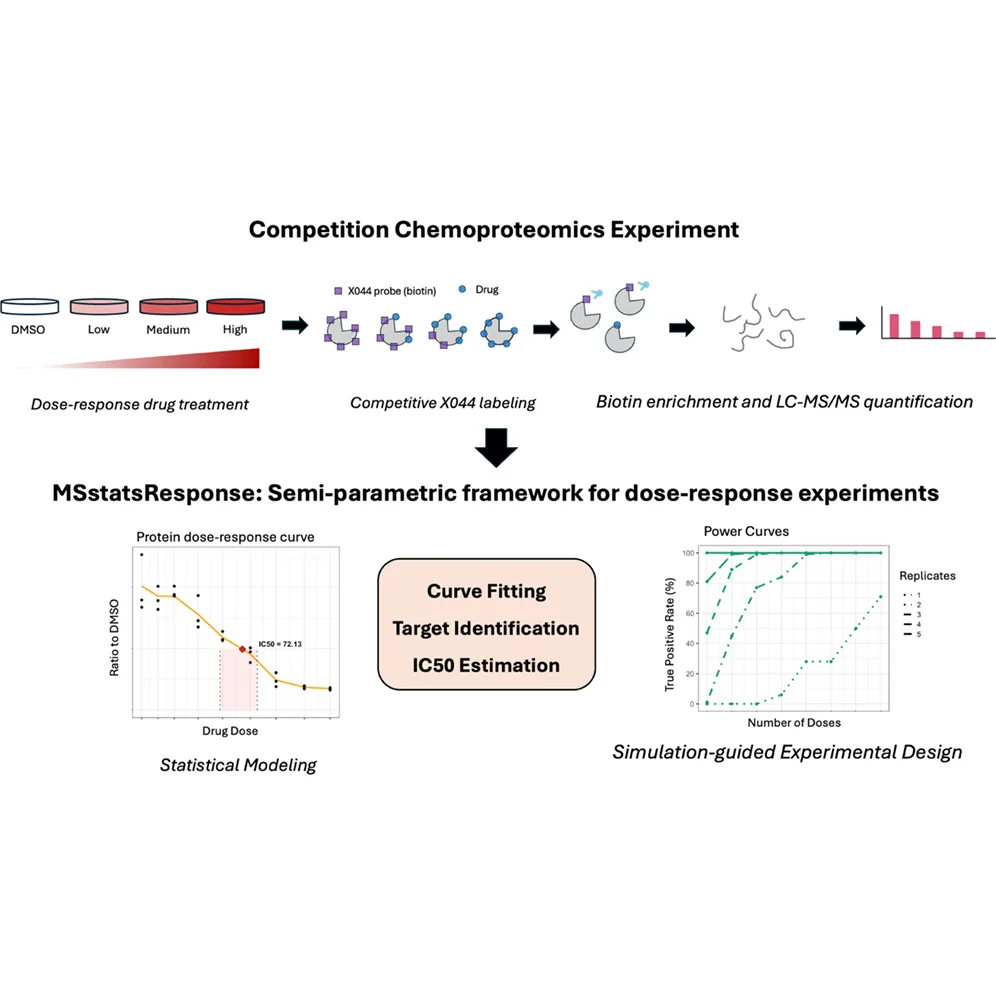

## In Brief

Here, we present MSstatsResponse, a semiparametric framework for analyzing chemoproteomic dose–response experiments. Using isotonic regression, MSstatsResponse models both increasing and decreasing monotonic responses. This improves detection of partial drug–protein responses without inflating false positives, and it preserves sensitivity to complete responses in experimental designs with fewer doses, where standard approaches often struggle. The framework also estimates IC50 values and provides a simulation-based approach to guide the choice of drug doses and biological replicate counts when planning future experiments.

Drug discovery characterizes drug–protein interactions using technologies such as CRISPR-based genetic screens (1, 2), thermal shift assays (3, 4), affinity-based profiling (5), and cellular imaging (6, 7). In recent years, chemoproteomics has emerged as a powerful and increasingly popular approach for mapping small molecule–protein interactions directly in biological systems (8, 9, 10). By integrating functionalized chemical probes with mass spectrometry (MS), chemoproteomics quantifies changes in protein abundance or engagement across a range of small molecule concentrations (8, 11, 12, 13, 14, 15). Chemoproteomic experiments have been applied in both basic and translational research to explore drug mechanisms (16), identify off-target effects (17, 18, 19), and prioritize lead compounds (20).

**Fig. 1:**
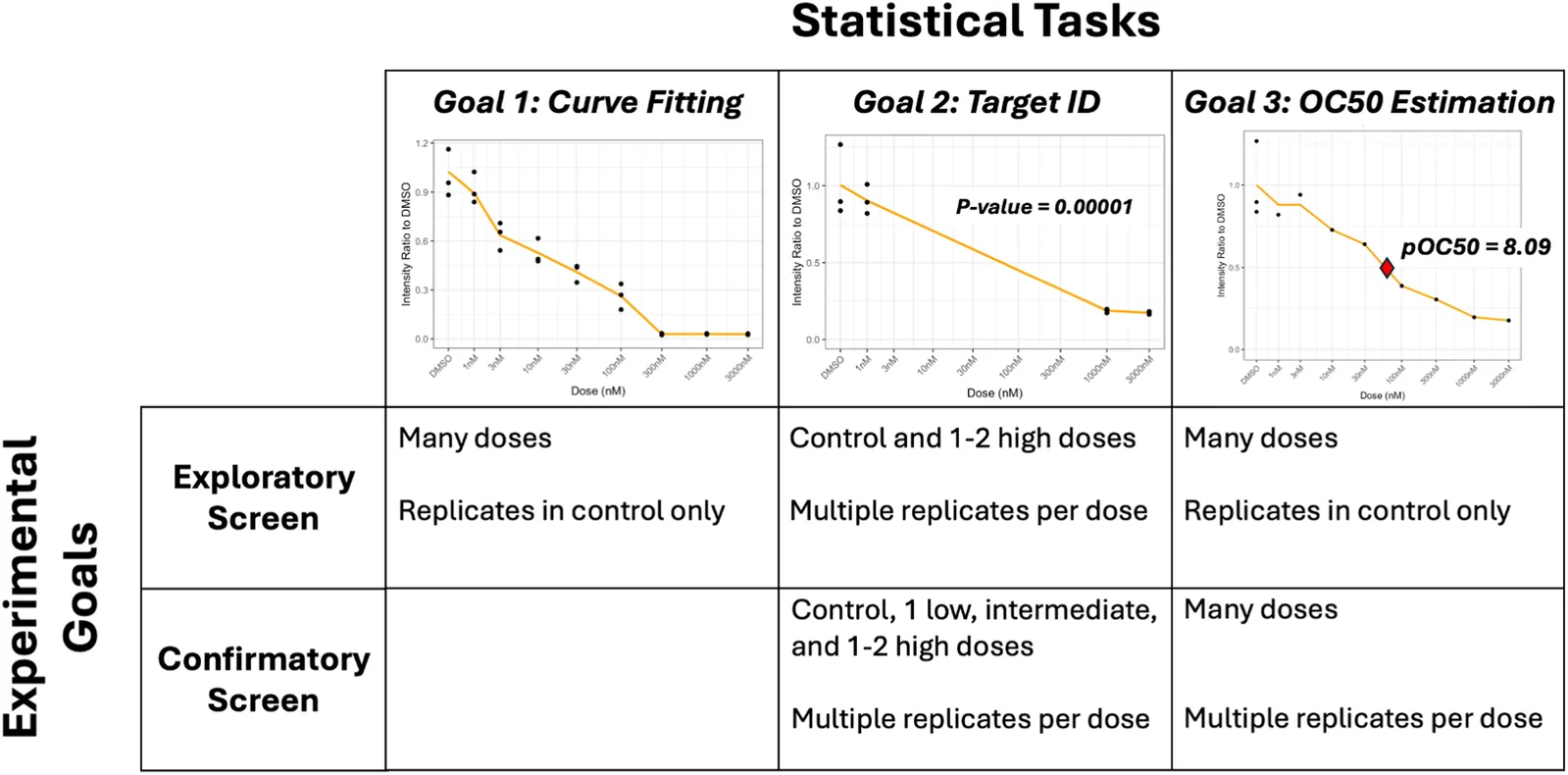
**The experimental design of a chemoproteomic experiment depends on the analysis objective.** Exploratory screens typically prioritize broad discovery and are optimized for detecting drug–protein interactions using a limited number of doses with more replicates. In contrast, confirmatory screens typically emphasize reproducibility and precision. Accurate OC50 (occupancy) estimation and curve fitting is achieved by including more doses and/or multiple replicates at each dose.

Experimental goals of MS-based chemoproteomic experiments range from exploratory screens profiling targets across many compounds, to confirmatory studies focused on estimating the dose–response curve and on figures of merit such as minimum inhibitory concentration, half effective/inhibitory/occupancy concentration (EC50/IC50/OC50 respectively), or toxicity thresholds (11, 21, 22, 23) (Fig. 1). In competition-based chemoproteomics, these “half-response” quantities are derived from changes in probe-bound protein abundance, and therefore reflect target engagement or occupancy with respect to the assay readout, rather than direct functional inhibition. A major consideration for these experiments is the choice of experimental design, which must balance the range and the number of doses and replicates. While single high-dose screens with biological replicates are common for target identification (24), single-replicate, multidose designs are favored for broad dose-range profiling (25).

Another consideration is the choice of spectral acquisition. For example, data-independent acquisition (DIA) is often preferred in exploratory settings where the identity of drug-responsive proteins is not known *a priori*, and broad proteome coverage enables unbiased detection of potential targets and off-targets (26). Targeted selected reaction monitoring (SRM), in contrast, is better suited for focused studies on a small, predefined protein set across a limited number of drugs (27). Tandem mass tag–based data-dependent acquisition (TMT-DDA) is another alternative for exploratory analyses, often chosen for its ability to reduce missing values within a single multiplexed set, but limited by its multiplexing capacity (typically 18 channels) (28).

Given the diversity of experimental goals, analysis tasks and resource allocations, there is a need for statistical frameworks that support experiments with both exploratory and confirmatory goals and diverse sources of uncertainty and variation, and provide valid statistical inference. Most existing statistical methods fit some form of monotonic curves to the dose–response profile of each protein, and derive figures of merit from these curves. The monotonic nature of the curve is meaningful in the context of chemoproteomics. For example, inhibitors and degraders typically exhibit nonincreasing profiles (i.e., decrease in abundance with high-dose treatment) (29, 30), while stabilizers and activators produce nondecreasing profiles (30, 31, 32). The curves often exhibit flat profiles reflecting saturation at both low and high doses, and a slope in the transition zone between the extremes. Therefore, existing curve-fitting packages such as drc (33), dr4pl (34), and nplr (35) fit four-parameter log-logistic sigmoid curves and provide parameter estimation and visualization. In many applications this mid-point parameter is reported as an IC50; in this article, for competition-based chemoproteomics, we refer to the analogous quantity as OC50 to emphasize that it is derived from probe-bound protein abundance and reflects target engagement/occupancy rather than direct functional inhibition. Despite their usefulness for curve fitting, these approaches lack formal statistical drug–target engagement detection, leaving researchers to manually review large numbers of curves. More recent CurveCurator (21) addresses this limitation of statistical inference, but relies on the same sigmoidal curve assumption. Such sigmoid curves become unstable in experiments with limited doses, replicates, and high variation, which may result in the loss of meaningful partial responses (i.e., monotonic dose-dependent changes that do not reach a complete response within the experimental dose range) (36, 37). Single-replicate experiments are particularly prone to introducing bias, inflating false-positives, and reducing reproducibility of the results (38).

To address these challenges of statistical analysis we introduce MSstatsResponse, a semiparametric statistical framework that accounts for the monotonicity of dose–response relationships but does not assume any parametric (e.g., sigmoid) form. Specifically, MSstatsResponse fits isotonic regression to protein-level abundance profiles that is compatible with all experimental goals, experimental designs, and MS acquisitions, as well as all statistical analysis tasks in Figure 1.

We evaluated the performance of MSstatsResponse by generating a benchmark data set that profiles the competition between the kinase-binding probe XO44 and the drug Dasatinib using DIA, TMT-DDA, and SRM, as well as on simulated data sets covering a range of experimental designs. Across these evaluations, MSstatsResponse consistently improved sensitivity, specificity, and reproducibility compared to the existing methods, particularly in experiments with limited data. For curve estimation, fewer doses were sufficient to capture meaningful differences in response profiles. For target identification, prioritizing biological replication over dose number improved the sensitivity of detecting drug–protein interactions. For OC50 estimation, incorporating replicates in the control condition and expanding the number of doses improved the precision of the estimation. Based on these results, we developed a practical simulation-based approach to select the appropriate number of doses and replicates in a future experiment for each experimental objective. MSstatsResponse is implemented as an open-source R/Bioconductor package that is freely available as part of the MSstats ecosystem for quantitative proteomics (39, 40, 41, 42, 43).

## Experimental Procedures

### Benchmark Experiments

A benchmark data set was generated by pretreated Jurkat cells with varying concentrations of a kinase inhibitor, Dasatinib, followed by treatment of a promiscuous kinase probe, XO44. Briefly, Jurkat cells were grown in RPMI (Thermo Fisher Scientific 11875-093) + 10% Fetal Bovine Serum (Thermo Fisher Scientific 16000044) + 1X Penicillin/Streptomycin (Thermo Fisher Scientific 15140148). Cells were pretreated with varying concentrations of Dasatinib for 1 h at 37 °C in serum-free media, followed by treatment of 2 μM XO44 for 30 min at 37 °C. After probe treatment, cells were spun down and washed with PBS to remove excess probe. Cells were lysed with PBS (14190-144) + 0.25% SDS (L4522)+ 1X HALT protease and phosphatase inhibitor (78442) + 1 μL benzonase (712063) followed by sonication (1 s pulse, 60% amplitude). A biotin handle for enrichment was installed via Click Chemistry (40 μM tris[(1-benzyl-1H-1, 2, 3-triazol-4-yl)methyl]amine (T2993), 1 mM tris(2-carboxyethyl)phosphine (20491), 1 mM CuSO4 (451657-10G), 10 μM biotin-TAMRA-azide) for 1 h at room temperature. Proteins were precipitated with acetone to remove click reagents, resuspended in PBS + 0.1% SDS, and probe-labeled proteins were enriched with high-capacity streptavidin beads (4 °C, 16 h). After enrichment, beads were washed with 3× PBS, 3x PBS + 0.1% SDS, 3× water. Beads were resuspended in 2 M urea (ZU10001) + 1 mM CaCl2 (21,098), proteins reduced with 10 mM DTT (D9163-1G), alkylated with 20 mM iodoacetamide (I11149-5G), and digested with trypsin (37 °C, 16 h, V5111). Following digestion, the peptides were desalted (The Nest Group, SNS SS18V) and split into three equal aliquots for the subsequent data acquisition methods. Cells were pretreated with Dasatinib so that the drug could engage its targets before the XO44 probe was introduced. The probe then labeled only the binding sites left unoccupied by the drug, such that increasing drug doses reduced probe-bound abundance in a concentration-dependent manner.

Positive controls SRC, ABL1, YES, CSK, LCK, and negative control EIF2AK4 were used for benchmarking performance (44, 45, 46). While Dasatinib is known to engage a broad range of kinases, only subsets were detectable in this data set. The benchmark experiment used a XO44-based enrichment strategy, which captured ATP-binding proteins accessible to the probe. The five targets were selected because they are kinases with well-documented, strong engagement within the experimental dose range across multiple ABPP platforms (44, 45, 46). EIF2AK4 was selected as a negative control by cross-referencing kinases enriched by XO44 with no dasatinib engagement at 100 nM or 300 nM (45) against an independent kinobeads data set in which it similarly showed no engagement (47). Response was measured across multiple drug doses and biological triplicates using three acquisition strategies: TMT-DDA, DIA, and SRM. More details on the experimental workflow can be found in Supplemental Sec. 1.

The raw MS data are publicly available on MassIVE under the accession numbers MSV000100970 (DIA dose-response data set), MSV000100968 (TMT dose–response data sets), and MSV000100969 (SRM dose–response data set). The intermediate data sets and analysis scripts used to reproduce the results in this manuscript are available on Zenodo at https://doi.org/10.5281/zenodo.18881723. All benchmark experiments were summarized to the protein level using the same tool (MSstats) to ensure a fair comparison. The same processing, normalization, handling of poor quality measurements, and missing value treatment were applied for every statistical method. A summary of each data set can be found in Table 1 and are described in further detail below.

**Table 1:** Data overview and availability

Metric	DIA	TMT	TMT-RTS	SRM	Simulation
No. of doses	9	6	6	9	2/4/5/9
No. of replicates	3	3	3	3	1/2/3/4
No. of proteins	7329	5935	6668	175	3000
Average no. of peptides	17	8	8.7	1	N/A
Positive controls	5	5	5	5	2000
Negative controls	1	1	1	0	1000
Data availability	MASSIVE	MASSIVE	MASSIVE	MASSIVE	Zenodo

“No. of doses” is the number of drug doses (concentrations) in each data set. “No. of replicates” is the number of replicates per drug dose; simulations were generated with varying replicate numbers. “No. of proteins” is the total number of proteins quantified. “Average no. of peptides” is the average number of peptide measured per protein. “Positive controls” are the five known Dasatinib targets (SRC, ABL1, YES, LCK, and CSK), and “Negative controls” is EIF2AK4. “Data availability” refers to the MassIVE repository ID for experimental data sets or the Zenodo repository for simulations.

#### Data Set 1: DIA benchmark

Samples were run on a Bruker timsTOF SCP using default acquisition parameters. The DIA data set comprises 27 samples, including triplicate dimethyl sulfoxide (DMSO) controls and eight drug doses (1 nM, 3 nM, 10 nM, 30 nM, 100 nM, 300 nM, 1 μM, and 3 μM). Data were processed using Spectronaut v18.6 in directDIA mode against a human reference proteome database from UniProt (20,375 sequences, downloaded on January 7, 2022) plus a contaminant database with the following relevant parameters: MZ Extraction Strategy set to maximum intensity, precursor Q value cutoff to 0.01, precursor PEP cutoff to 0.01, protein Q value cutoff to 0.01, fixed modifications (C Carbamidomethyl), variable modifications (protein N-term acetylation and M oxidation), enzyme set to trypsin/P with up to two missed cleavages, and mass tolerance strategy set to dynamic with correction factor of 1.

#### Data Set 2: TMT Benchmark

The desalted peptides were dried down and resuspended in 100 mM 4-(2-hydroxyethyl)piperazine-1-propanesulfonic acid, pH 8.5 (Thermo Fisher Scientific, J61476.AE) at a final concentration of 30% acetonitrile and were reacted with 125 μg of TMTpro reagent for 1 h at room temperature. The TMT reaction was quenched with the addition of ammonium hydroxide to a final 0.5% concentration. The samples were then pooled and desalted and the TMT-labeled peptides were fractionated using the Pierce High pH Reversed-Phase Peptide Fractionation Kit (Thermo Fisher Scientific 84868) according to manufacturer’s instructions. LC-MS (3) analysis of the TMT samples was performed on a Dionex Ultimate 3000 UPLC system (Thermo Fisher Scientific), coupled online to an EASYSpray ion source and an Eclipse Tribrid mass spectrometer with FAIMS (Thermo Fisher Scientific). TMT-labeled peptide fractions were separated on an EASYSpray C18 column (75 μm × 50 cm, ES903) heated to 50 °C using mobile phases A (0.1% formic acid in water) and B (0.1% formic acid in 90% acetonitrile) at a flow rate of 250 nl/min with a linear gradient from 8% B to 28% B for 117 min followed by a ramp to 35% B over 10 min. The real-time search (RTS) data was acquired using previously described instrument settings (48). The SPS MS3 data set was acquired using identical instrument settings but without the RTS step.

The TMT data were searched in Proteome Discoverer v 3.0 against a human reference proteome database from UniProt (20,526 sequences, downloaded on February 13, 2024) plus a contaminant database. The Proteome Discoverer workflow utilized the Spectrum Files recalibration node for offline mass recalibration and then required 5 ppm mass accuracy for the precursor ions and 0.5 Da mass accuracy for the fragment ions for the Sequest HT search. Static modifications were set for carbamidomethyl modifications to cysteine and TMTpro on peptide N termini and lysine residues. Dynamic modifications were set for oxidation of methionine, deamidation of asparagine, and methionine loss plus acetylation of protein N termini. Matched spectra were scored by Percolator and reporter ion signal-to-noise values were extracted. Parsimonious protein assembly at a 1% false discovery rate for proteins and peptides was conducted and reporter ion quantitation was performed on protein unique peptides only. Additional filters were applied to ensure data quality, only peptides without variable modifications, which also had an average signal-to-noise of at least 10, a precursor co-isolation threshold of ˂30%, and a synchronous precursor selection match of at least 70% were used.

#### Data Set 3: SRM Benchmark

SRM was performed using a TSQ Altis Mass Spectrometer with an EASY-Spray Ion Source (Thermo Fisher Scientific) coupled with a Dionex UltiMate 3000 UHPLC (Thermo Fisher Scientific). Peptides were first trapped on a C18 trap cartridge (#174500, Thermo Fisher Scientific) and washed with 0.5% TFA for 2 min. Then, they were separated on an EASY-Spray column (#ES900, Thermo Fisher Scientific) at a flow rate of 400 nl/min over a linear gradient: 1 to 45% B, 2′-25′ (solvent A: 2% acetonitrile 0.1% formic acid, solvent B: 90% acetonitrile 0.1% formic acid). Subsequently, the trap cartridge and analytical column were each washed with 80% acetonitrile 0.1% formic acid and equilibrated in either 0.5% TFA or 1% A, respectively, prior to the next injection. The column temperature was maintained at 50 °C. Peptides were ionized at a spray voltage of 2.8 kV in positive ion mode while the ion transfer tube temperature was maintained at 300 °C. SRM was carried out on 175 proteins, using 1 peptide per protein and two precursor ions for each peptide (1 light, 1 heavy). Full SRM table can be found in Supplemental Information (Supplemental Table S1). Key parameters included scheduled acquisition with a 90 s retention time window, a cycle time of 2 s, Q1 resolution (full width at half maximum) of 0.7, and Q3 resolution (full width at half maximum) of 1.2. After data were acquired, peak area integration was conducted using Skyline (64 bit) 23.1.0.268. The SRM data set includes 27 samples, consisting of triplicate DMSO controls and the same eight drug doses as the DIA data set (1 nM to 3 μM).

### Simulated Data Sets

To evaluate method performance under different experimental designs and conditions, we simulated chemoproteomic data sets based on template protein abundances from the DIA benchmark (Supplemental Fig. S3). Template proteins were defined as showing a complete or partial response to drug treatment (true positives) or no response (true negatives). A complete response corresponded to proteins whose probe-bound abundance decreased to near zero at the highest drug dose. A partial response corresponded to proteins whose probe-bound abundance decreased to approximately 50% of their baseline level. Proteins with no response maintained constant abundance across all doses, indicating no drug effect. Specifically, the SRC protein served as a complete-response template, TEC as a partial-response template, and EIF2AK4 as a nonresponding template. We then generated multiple experimental scenarios by varying the number of doses, biological replicates, and amount of variation to benchmark each method across a range of design settings. Full simulation details are provided in the Supplemental Sec. 1.2 and code on Zenodo at https://doi.org/10.5281/zenodo.18881723.

## Background

### Input Data and Notation

Statistical analyses of chemoproteomic experiments typically take as input protein-level abundance values. For acquisitions such as DIA, DDA, SRM, or PRM, the spectral features are first processed with pipelines such as Skyline (49), Spectronaut (50), FragPipe (51), Proteome Discoverer (52) or MaxQuant (53) that detect, identify and quantify spectral features and summarize the feature abundances into a single value per protein per run. Alternatively, specialized packages such as MSstats (40), QFeatures (54), MaxLFQ (55), or MSnbase (56) take as input the identified and quantified spectra features and provide dedicated statistical frameworks for protein summarization and quality control. Important aspects of protein-level summarization include normalization of feature or protein abundances between MS runs and treatment of missing values.

### Existing Statistical Methods for Dose–Response Analysis

In this article, we consider a protein measured in an experiment with *r* = 1, …, *R* biological replicates at each of the *d* = 0, 1, …, *D* doses, where *d* = 0 denotes a control such as DMSO. Therefore, the total number of measurements is *R*(*D* + 1). Let *x*_*d*_ denote the drug dose at index *d*, and *a*_*dr*_ denote the corresponding protein abundance in replicate *r* on the original (i.e., not log-transformed) scale.

The most popular model for dose–response analysis in chemoproteomics is the four-parameter log-logistic model (4PL), also known as the Hill model, implemented in tools such as drc (33), dr4pl (34), and CurveCurator (21). These models assume a sigmoidal response as summarized in Table 2. From the curve fit, the tools estimate the lower and upper asymptotes, slope (Hill coefficient), and OC50 parameters as detailed in Supplemental Table S1.

**Table 2:** Curve fitting models and input protein abundance scales of the existing and proposed approaches

Method	Input	Model
drc	Ratio to DMSO (Equation 1); original scale	$y_{dr}=\theta_{1}+\frac{\theta_{4}-\theta_{1}}{1+10^{\theta_{3}\left(x_{d}-\log_{10}\theta_{2}\right)}}+\epsilon_{dr}$ ${\epsilon_{dr}}_{∼}^{iid}N\left(0,\sigma^{2}\right)$
dr4pl		
CurveCurator	Ratio either to DMSO (Equation 1) or to abundance at min(*x*_*d*_); original scale	
ANOVA	log_2_ scale	$y_{dr}^{\prime }=\mu +x_{d}+\epsilon_{dr}$ $\sum_{d=0}^{D}x_{d}=0;\;{\epsilon_{dr}}_{∼}^{iid}N\left(0,\sigma^{2}\right)$
MSstatsResponse	Curve fitting and OC50 estimation: ratio to DMSO (Equation 1); original scale. Target identification: log_2_ scale	$y_{dr}^{\prime }=f\left(x_{d}\right)+\epsilon_{dr}$${\epsilon_{dr}}_{∼}^{iid}N\left(0,\sigma^{2}\right)\;where$ $f\left(x_{d}\right)>f\left(x_{1}\right)>\cdots >f\left(x_{D}\right)$

#### drc

This R package fits and visualizes dose–response curves. It is not specifically designed for chemoproteomics experiments, but can easily be applied to any data set that quantifies dose–response relationships. Prior to curve fitting, users must transform the input protein abundances at each dose and replicate relative to the control, that is,

(1) $$y_{dr}=\frac{a_{dr}}{{\bar{a}}_{0}},d=0,\ldots ,D;\;r=1,\ldots ,R$$

where ${\bar{a}}_{0}$ is the mean protein abundance in the control. This transformation rescales the data such that in a 4 PL, the response is expressed as a percent reduction relative to the control (i.e., 0–100%). Parameters of the curve are then estimated by minimizing the sum of squared residuals. For each of the four model parameters (i.e., lower and upper asymptote, slope, and mid-point concentration), the package reports the estimated values along with the fitted sigmoid curve.

While drc addresses curve fitting and OC50 estimation tasks, it does not provide a formal hypothesis testing framework for identifying significant drug–protein interactions. The package provides standard errors and *p* values for each of the four model parameters, and tests the null hypothesis of each parameter equal to a certain value (e.g. slope = 0); however, it does not assess the overall curve fit. As a result, many users choose to manually inspect pages of dose–response curves, or apply filtering based on slope and OC50 estimate specific *p* values, to find drug–protein interactions.

#### dr4pl

Developed as an alternative to drc, this R package addresses several of its limitations (in particular, model fitting convergence issues) to provide a more robust framework. Like drc, it is not designed specifically for chemoproteomics experiments, but can be applied with similar data preparation. In practice, it takes as input protein abundances transformed as in Equation 1, and fits and visualizes a 4 PL curve to estimate the mid-point concentration. Like drc, dr4pl returns 95% confidence intervals (CIs) for all model parameters (Supplemental Table S1). However, this R package does not improve on the hypothesis testing task for identifying significant drug–protein interactions. Users must define their own criteria for identifying interacting proteins based on model outputs, such as checking whether the CI for the slope contains zero, or whether the CI for OC50 lies within the experimental dose range.

#### CurveCurator

This Python package was developed specifically for chemoproteomics-like experiments and integrates normalization, curve fitting, and statistical inference into a single workflow. Unlike drc and dr4pl, it takes as input summarized protein abundances, *a*_*dr*_. Within the workflow, it automatically applies the transformation in Equation 1, and provides alternative transformations relative to the smallest drug dose (or another chosen control). Upon the transformation, it facilitates optional steps such as global median centering, imputation of missing values using intensity-based rules (e.g., imputing low-intensity values from a distribution), and filtering out curves with excessive number of missing values.

CurveCurator fits the same 4 PL as drc and dr4pl and provides single-point OC50 estimates. In contrast to the other methods, it implements a formal hypothesis test for drug–protein interaction detection by comparing each fitted curve to a null model of constant response with a recalibrated F-statistic. This recalibration uses simulated null curves to estimate effective degrees of freedom and *p* values. Beyond the *p* values, CurveCurator combines statistical significance with effect size (log2 fold-change between lowest and highest dose) into a single relevance score. Curves are then classified as significantly upregulated, downregulated, not regulated, or unclear (for high-variance cases).

### Existing General Statistical Methods for Proteomic Experiments

All the packages above transform the input protein abundances at each dose and replicate relative to a control. While fitting the curve on this scale helps visual assessment and enables direct estimation of OC50, this transformation has negative consequences. For example, if the variability in the DMSO condition is not properly estimated (e.g., due to lack of replicates), the resulting curves shapes may become distorted. Alternatively, if variability is high within the control replicates, then that noise is propagated through all other measurements. Therefore, it is attractive to consider alternative statistical analyses that do not require such transformation.

#### ANOVA

ANOVA is a general statistical approach implemented in most programming languages. It is not specifically designed for proteomics or dose–response experiments, but is easily applied. In the context of proteomics, it translates to a test of differential protein abundance across dose conditions. Similarly to drc, dr4pl, and CurveCurator, it takes as input protein abundance values; however, the ratio-scaled values are not required. Instead, ANOVA compares the means of log_2_-transformed protein abundances at each *D* + 1 doses.

(2) $$y_{dr}^{\prime }=\log_{2}\left(a_{dr}\right),d=0,1,\ldots ,D;\;r=1,\ldots ,R$$

ANOVA requires at least two replicates for at least 1 dose; however, designs with replicates at each dose perform best. The ANOVA model assumes that each dose has its own expected value of protein abundance and an equal variance of replicates for each dose, including for the control such as DMSO (Table 2). Parameters of the model are estimated using least squares. Hypothesis test compares the means of protein abundance at each dose to the null curve with a constant response using F-test (Supplemental Table S1).

Since ANOVA avoids taking ratios of protein abundances relative to a control, it is not susceptible to the artifacts of this approach. Moreover, ANOVA does not assume any functional relationship between the doses and the response, providing additional flexibility. However, this flexibility has its own drawbacks. In particular, users have to manually format protein abundances, manually perform normalization and missing value imputation if needed, and interpret the ANOVA output. Furthermore, the fitted profiles can exhibit nonmonotonic hook-like patterns that can reflect either true biological signal or single-dose technical outliers. While ANOVA is better suited for data sets where nonmonotonic responses are expected, this same flexibility makes it more sensitive to noise. The assumption of constant variance between DMSO and drug-treated protein abundances may not hold. Finally, ANOVA does not naturally support the estimation of OC50.

#### MSstats

MSstats is a family of R packages developed for statistical analysis of MS-based proteomics data (40, 57). MSstats supports input from all major MS acquisitions such as DDA and TMT-DDA, DIA, and SRM, and includes converters for many widely used spectral processing tools such as Skyline (49), Spectronaut (50), FragPipe (51), Proteome Discoverer (52), and MaxQuant (53) that identify and quantify spectral features. The processing workflow supports steps for quality control, multiple normalization strategies (e.g., median, reference-based, global), optional imputation for missing values using an accelerated failure time model, and protein summarization from peptide- or fragment-level data using Tukey’s Median Polish. Therefore, MSstats can be applied to data sets generated from a range of experimental questions and pipelines, and supports the reproducibility of downstream statistical analyses (57).

MSstats log-transforms feature intensities prior to summarization, and therefore protein-level summaries are comparable to the log_2_ scale. Statistical analyses in MSstats model these protein-level abundances, and extend ANOVA using a flexible family of linear mixed-effects models applicable to complex designs. In dose–response experiments, MSstats supports comparisons of specific doses (e.g., DMSO *versus* the maximum dose) using user-defined contrasts (58). Although MSstats is better suited for chemoproteomic experiments than ANOVA by automating the workflow and providing more flexible models, the available statistical analyses have the same drawbacks as ANOVA and currently do not support all the tasks necessary for chemoproteomics.

## Results

### Overall Workflow in MSstatsResponse

**Fig. 2:**
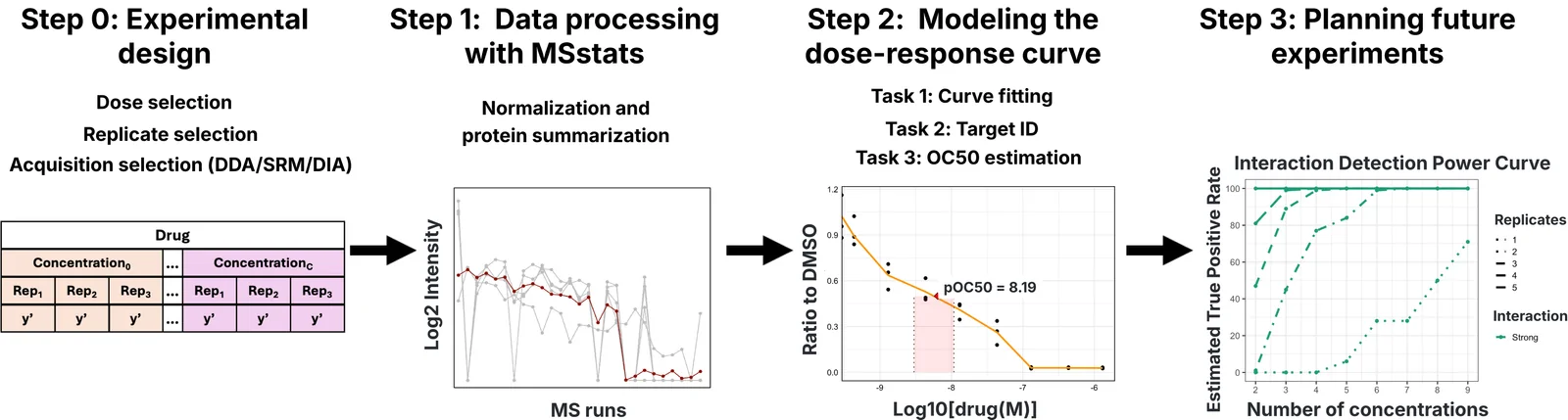
**Proposed workflow for chemoproteomic experiment data analysis with MSstatsResponse.** Step 0: the workflow starts with the experimental design, which includes selection of doses, replicates, and acquisition. Step 1: the acquired data are normalized, and protein abundances are summarized using MSstats. Step 2: MSstatsResponse supports three analysis tasks: curve fitting, target identification, and OC50 estimation. Step 3: the existing data set serves as a basis for selecting an appropriate number of doses and replicates in future investigations.

The proposed approach implemented in MSstatsResponse is overviewed in Figure 2. The MSstatsResponse is implemented in an open-source R package fully integrated with the MSstats ecosystem, available at https://bioconductor.org/packages/MSstatsResponse.

#### Step 0: Experimental design

Since different analysis goals require different trade-offs between the number of drug doses and biological replicates, MSstatsResponse supports all such designs. Exploratory screens focused on target identification (i.e., detection of proteins whose abundance changes in response to drug treatment, including on-target, off-target, and downstream effects) typically use a single or few doses with replication, while those aimed at curve fitting or OC50 estimation may instead prioritize dose coverage with fewer replicates. Confirmatory studies generally benefit from both more replicates and broader dose coverage. MSstatsResponse also supports unreplicated experiments for curve fitting, OC50 estimation, and maximum log2 fold-change estimation; however, evaluating the statistical significance of target identification requires replicates in at least 1 dose.

#### Step 1: Data processing with MSstats

To optimize this step for quantitative proteomics, MSstatsResponse builds directly on the MSstats infrastructure for preprocessing, normalization, and protein summarization. MSstats provides the option of global median normalization, appropriate when the drug is expected to affect only a small number of proteins. If the drug is expected to affect many proteins, median normalization should be turned off, and the data should be normalized with respect to reference protein standards or be left without normalization. MSstats excludes proteins with an excessive number of missing values and imputes the remaining values using an accelerated failure time model (59, 60). The resulting protein-level summaries serve as input for all subsequent MSstatsResponse analyses. However, using MSstats is not required, and the remaining steps are in principle compatible with other appropriate data processing and protein summarization methods and software.

#### Step 2: Modeling the Dose–Response Curve

This step is the main contribution of this manuscript. Similarly to ANOVA and MSstats, we propose to analyze protein-level summaries on the log-transformed scale (as opposed to the ratio-transformed scale). Similarly to drc, dr4pl, and CurveCurator, MSstatsResponse includes a monotonic relationship constraint between the dose and the response to avoid overfitting to experimental artifacts. However, the monotonic relationship is not restricted to a strict sigmoid shape.

To fit such a curve, MSstatsResponse relies on isotonic regression (Table 2) (61, 62). Isotonic regression is a nonparametric statistical model that specifies a piecewise linear function under a monotonicity constraint. Its utility has been demonstrated in dose-finding in clinical trials (63), high-throughput genotoxicity screening (64), and causal dose–response estimation (65). In these applications, isotonic regression was favored for its greater flexibility as compared to parametric curves, and its robustness in fitting nonsigmoidal curve shapes. However, its utility for chemoproteomics has not been explored so far. MSstatsResponse relies on the robustness of isotonic regression to irregular or noisy patterns and on its ability to easily accommodate various experimental designs and analysis tasks. We also considered alternative options, such as a monotone I-spline (66), but ultimately opted for the simpler isotonic regression model. These smooth monotonic estimators can introduce additional complexity, including specifying a knot structure and added degrees of freedom that can be difficult to control against per-protein overfitting (58). The three primary analysis tasks (curve fitting, target identification, and OC50 estimation) are described in more detail below.

#### Task 1: Curve fitting

By default, MSstatsResponse specifies a nonincreasing constraint, reflecting the expected trend in inhibitor screens, but this can be easily reversed to model nondecreasing responses when appropriate. Under the nonincreasing constraint, the method minimizes the weighted squared error between the observed protein abundances and a function *f*(*x*_*d*_) (61, 62).

(3) $${\hat{y}}_{dr}^{\prime }={\mathrm{argmin}}_{f\in F}\sum_{d=0}^{D}\sum_{r=1}^{R}w_{dr}{\left(y_{dr}^{\prime }-f\left(x_{d}\right)\right)}^{2}$$

where F is the set of all piecewise linear functions that satisfy the constraint, and *w*_*dr*_ are optional observation weights.

Parameters of *f*(*x*_*d*_) are estimated using the pool-adjacent-violators algorithm (PAVA) (61, 62). In an unreplicated experiment (*R* = 1), PAVA begins with the observed abundances at each dose *d*, $y_{d1}^{\prime }$, as the fitted values. It then scans through adjacent dose levels and checks whether the monotonicity constraint is violated, that is,. whether $y_{d+1\;r}^{\prime }>y_{d1}^{\prime }$, to ensure a nonincreasing fit. If the monotonicity constraint is violated, PAVA iteratively pools adjacent dose levels until the constraint is satisfied. This process continues until all adjacent pairs satisfy the monotonicity constraint. The resulting fit is a piecewise linear, nonincreasing function. In replicated experiments (*R* > 1), the initial fitted values are replaced by the mean abundances at each dose ${\bar{y}}_{d\cdot }^{\prime }$. The default in MSstatsResponse is set to equal weights. In experiments with unequal weights, pooled values are replaced with their weighted averages. This makes the model directly compatible with external quality scores. For example, the per-measurement quality scores produced by MSstats+ (67) can be supplied as observation weights *w*_*dr*_, to down weight low-quality measurements in the fit.

Isotonic regression is compatible with both log-transformed and ratioed measurements. Therefore, MSstatsResponse uses different scales depending on the analysis task. For curve fitting and visualization, MSstatsResponse models the data on the ratioed scale, since this aligns with the familiar convention of expressing dose–response relationships as a percent change relative to control. Users also have the option to visualize fitted curves on the log scale (Supplemental Fig. S5). In competition-based chemoproteomics, this corresponds to the fraction of probe-bound protein remaining as a function of drug concentration. Since protein-level abundances reported by MSstats are comparable to the log_2_ scale, the task begins by exponentiating the summaries to recover the original-scale intensities, that is, $a_{dr}=2^{y_{dr}^{\prime }},d=0,1,\ldots ,D,R=1,\ldots ,R$. These values are then normalized relative to the control condition as in Equation 1, yielding the ratio-scale responses *y*_*dr*_. In all cases, the *x*-axis (drug dose) is transformed to log_10_ M units, following the guidance in (36).

#### Task 2: Target identification

For target identification (i.e., detection of proteins whose abundance changes in response to drug treatment, including on-target, off-target, and downstream effects), MSstatsResponse fits the isotonic regression on the log-transformed (as opposed to ratioed) scale to better match the assumptions of the downstream statistical inference. To detect drug–protein interactions, MSstatsResponse tests the null hypothesis of constant protein abundance across all doses, that is, $H_{0}:y_{dr}^{\prime }=\mu +\epsilon_{dr},\epsilon_{dr}\overset{iid}{∼}N\left(0,\sigma^{2}\right)$ *versus* the alternative $H_{a}:y_{dr}^{\prime }=f\left(x_{d}\right)+\epsilon_{dr},\epsilon_{dr}\overset{iid}{∼}N\left(0,\sigma^{2}\right)$, where *f*(*x*) is as in Equation 3 and *d* = 0, 1, …, *D*, *r* = 1, …, *R*, using a standard F-test (Supplemental Table S1). Approximate *p* values are reported from an isotonic regression-based F-test comparing these two models. Because the isotonic fit is constrained and its number of pieces is estimated from the data, its effective degrees of freedom are not fixed as in ordinary regression. Referencing this statistic against a standard F-distribution therefore does not produce a uniform null distribution. Instead, null simulations show the resulting *p* values are conservative, and they should be interpreted as approximate, conservative *p* values for detecting monotone dose–response behavior (Supplemental Fig. S6).

The log transformation $y_{dr}^{\prime }$ for this step was chosen based on methodological considerations, specifically on the fact that dividing all the observed values by a common observed reference violates the assumption of independence of the transformed values. The choice was also confirmed empirically through simulations. Supplemental Fig. S7 illustrates that the log scale increased the sensitivity of detecting true interactions without inflating the false positives as compared to the ratioed scale, notably for proteins with partial drug responses.

#### Task 3: OC50 estimation

In this article, we define OC50 as the drug concentration at which the probe-bound protein abundance is reduced to 50% of its control level. In a competition-based chemoproteomics assay, this corresponds to the dose at which half of the probe-binding capacity has been competed away. Since the curves on the ratioed scale have an intuitive interpretation as percent change relative to control (36), MSstatsResponse estimates OC50 on the ratioed scale as in task 1.

Since the isotonic regression curve *f*(*x*_*d*_) is piecewise linear, MSstatsResponse linearly interpolates between the values *f*(*x*_*d*_) > 0.5 and *f*(*x*_*d*+1_) < 0.5 that are closest to 0.5.

(4) $$\text{OC}50=\frac{x_{d+1}\left(f\left(x_{d}\right)-0.5\right)+x_{d}\left(0.5-f\left(x_{d+1}\right)\right)}{f\left(x_{d}\right)-f\left(x_{d+1}\right)}$$

The parameter is estimated using fitted values $\hat{f}\left(x_{d}\right)$. If all $\hat{f}\left(x_{d}\right)>0.5$, the OC50 is labeled as undefined. Unlike sigmoid-based methods such as in drc and dr4pl, which can extrapolate an OC50 and its CI beyond the measured doses, MSstatsResponse only estimates OC50 within the observed dose range and otherwise reports NA. A similar approach can estimate other figures of merit such as OC25, OC95, etc. By default, OC50 is defined relative to the control baseline (the dose at which the response reaches 50% of control). For cases where the half-maximal point relative to the fitted response range is of interest, MSstatsResponse also provides an option to estimate the EC50, defined as the dose at which the response reaches the midpoint between the fitted minimum and maximum.

All OC50 values in MSstatsResponse are reported as pOC50 (i.e., −log_10_OC50) values. This follows standard reporting practice in the field (36), where larger values indicate higher potency. For simplicity, we refer to pOC50 values as OC50 throughout the text.

CIs associated with the estimated OC50 are obtained using stratified bootstrap resampling (default *n* = 1000). To preserve the replicate structure of the experiment the procedure resamples the observations separately within each dose, estimates OC50 from each resampled data set, and reports the quantiles of the empirical distribution of resampled estimates.

Although MSstatsResponse estimates OC50 on the ratio-transformed scale, the approach is in principle compatible with the log scale as described in Supplemental Table S1. Supplemental Fig. S8 illustrates that the resulting estimates and CIs are similar on both scales.

#### Step 3: Planning Future Experiments

Statistical experimental design evaluates the performance of a future experiment with various combinations of doses and replicates, and given realistic estimates of systematic and random biological and technological variation. MSstatsResponse implements functionalities for such experimental design, treating the current data set as representative of future investigations.

First, MSstatsResponse creates systematic dose–response profiles representative of future complete, partial, and nonresponding proteins. Users specify the unique identifiers (e.g., protein IDs) corresponding to these representative proteins, which are selected from the fitted dose–response curves in the current data set. The tool can use 1, 2, or all 3 response types as templates, depending on the user’s goals. MSstatsResponse then simulates future experimental observations by adding instances of random variation to the systematic profiles as described in (Supplemental Algorithm 1). Each simulated data set can then be analyzed for (1) target identification (complete *versus*. partial *versus*. nonresponding proteins), or (2) OC50 estimation. For OC50 estimation, MSstatsResponse provides the option to estimate OC50 values for each simulated protein and, optionally, to compute CIs for these estimates.

To ensure accurate results, each combination of replicates and doses is repeated in 1000 simulated data sets. For each combination, MSstatsResponse reports key performance metrics, including average estimated true positive rates for complete and partial responses and average false-positive rates for noninteracting proteins. For OC50 estimation, it reports parameter estimates, and optional CIs for all simulated proteins. The results are summarized in plots such as in Figure 2, visually trading-off doses and replicates.

### Limitations

MSstatsResponse also has limitations specific to each of the three analysis tasks, which we outline below. For curve fitting, the method’s monotonicity assumption means it is not well suited to fitting nontraditional curve shapes such as hook effects or biphasic curves. For target identification, the method requires replicates in at least one dose to calculate *p* values, and because the isotonic fit’s effective degrees of freedom are not fixed, the resulting *p* values are approximate and conservative. For OC50 estimation, the method does not extrapolate beyond the observed dose range, so it returns not available when the response does not reach 0.5.

## Evaluation

### Evaluation Strategy

We compared the performance of MSstatsResponse to that of dr4pl, CurveCurator, and ANOVA. We omitted drc as this older method is relatively similar to dr4pl but less sensitive in drug target identification (Supplemental Table S2). Analyses were performed on both experimental and simulated data sets to assess method performance under confirmatory and exploratory experimental designs (Fig. 1). Confirmatory experiments were represented by the full benchmark data sets, each with nine doses and three replicates. Exploratory single-replicate experiments were represented by separately analyzed individual replicates from these data sets. Additional analyses generated intermediate designs with varying numbers of doses and replicates.

Each method was evaluated with respect to its ability to address the three analysis tasks in Figure 1. Task 1 (curve fitting) was visually assessed by plotting dose–response curves. Task 2 (target identification) was evaluated with respect to the performance of hypothesis testing. We chose significance thresholds that we believed to be most comparable across methods. Specifically, dr4pl did not provide *p* values, but provided parameter estimates and the 95% CIs for all four model parameters: upper asymptote, lower asymptote, slope, and OC50 value. We defined significant drug–protein interactions based on the following criteria: (1) OC50 95% CI is within experimental dose range, and (2) slope is decreasing and 95% CI does not include 0. Evaluations tested using only criteria (1) were overly sensitive and produced variable results across data sets (Supplemental Fig. S9). For CurveCurator, we determined drug–target interactions using relevance scores, and users-defined significant thresholds. We defined significance cutoffs as curve fold-change: 0.5 and false discovery rate-adjusted *p* value of *q* = 0.05, and kept these thresholds fixed across all experimental, single-replicate, and simulated analyses. For ANOVA and MSstatsResponse, evaluations were done at an false discovery rate-adjusted *p* value cutoff of *q* = 0.05.

Evaluation on the experimental data sets used established Dasatinib targets as positive controls. Since most proteins measured by the XO44 enrichment workflow are expected to reflect background rather than true drug engagement, hits were reported both as the total number of significant proteins and the total number of significant kinase proteins. Evaluation on the simulated data sets was reported in terms of true positive rates $\frac{TP}{TP+FN}$ for simulated complete and partial responses and false-positive rates $\frac{FP}{TN+FP}$ for noninteractors.

Task 3 (OC50 estimation) was evaluated based on the precision of the estimated OC50 values. Precision was measured by the width of their 95% CIs. Consistency of the estimates across different experimental designs was also used to evaluate reproducibility.

### All Acquisition Strategies Were Suitable for Chemoproteomics Experiments

All three acquisition strategies in this study, DIA, TMT, and SRM, produced high-quality data suitable for chemoproteomics experiments. All five known Dasatinib targets demonstrated the expected response patterns (Supplemental Fig. S10). While proteome coverage differed across methods (Table 1, Supplemental Fig. S2), these differences did not substantially limit the detection of drug–protein interactions. The choice of acquisition strategy is most important when considering protein coverage and alignment with the experimental goal. Our benchmark results further indicated that study design and statistical model choice had a greater impact on sensitivity and reproducibility than the acquisition method itself. Specifically, the number of replicates, the selection of drug doses, and the robustness of the statistical model had a much larger influence on performance. These considerations are evaluated in detail in the following sections. We use the DIA benchmark data set as the primary example in the following sections. Results for TMT and SRM benchmarks are provided in the Supplemental Sec. 4.

### In Experimental Data Sets, Statistical Methods Produced Distinct Dose–Response Curves

Curve-fit visualizations differed across statistical approaches. dr4pl and CurveCurator both fit 4 PL and therefore produced nearly identical sigmoidal curves. MSstatsResponse only specified a monotonic nonincreasing constraint, which made it robust to nonsigmoidal shapes and resistant to outlier-driven distortions. In contrast, ANOVA modeled each dose independently, which often yielded jagged fits and overfitting to outliers.

**Fig. 3:**
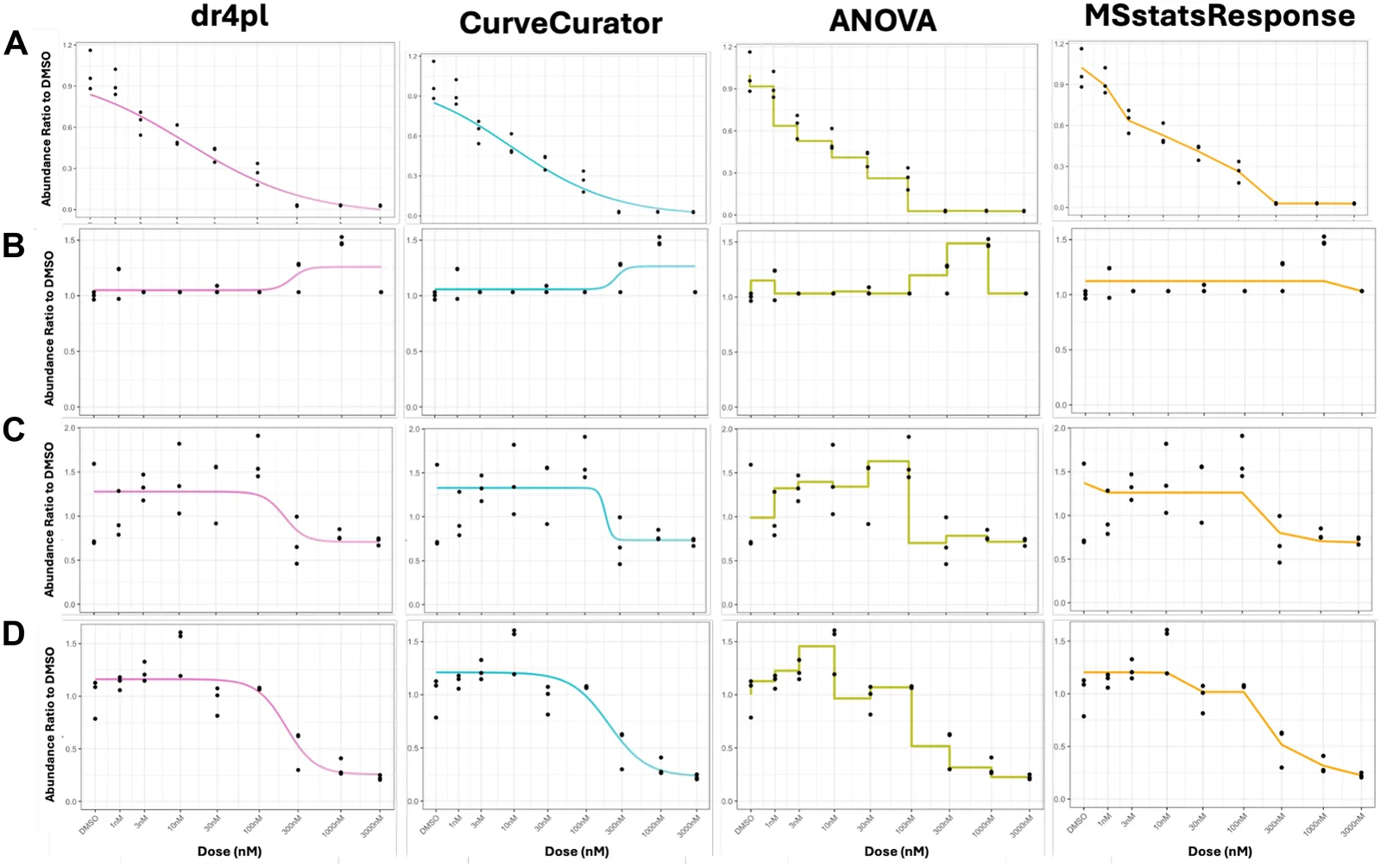
**DIA benchmark: examples of fitted dose–response curves across methods.***Rows*: proteins. *Columns*: methods/*dots*: input protein abundances. *Lines*: fitted curves. *A*, a clear sigmoidal decrease where all methods agree. *B*, a flat response with a high-dose uptick. dr4pl and CurveCurator impose an increasing sigmoidal shape, ANOVA overfits the outlier, and MSstatsResponse remains flat. *C*, a noisy profile at low doses with a decrease at higher doses. All methods capture the overall decline except ANOVA, which overfits to the increase. *D*, increase at low dose, then decrease at higher doses. MSstatsResponse remains flat through the rise and transitions downward only once the data support a decline. DIA, data-independent acquisition.

Figure 3 illustrates these behaviors. Row 1 (SRC, positive control) shows a clean sigmoidal decrease, where all methods agree. Row 2 (CD3G) is flat with a high-dose uptick; dr4pl and CurveCurator forced an increasing sigmoidal shape, ANOVA exaggerated the outlier, and MSstatsResponse maintained a flat, nonincreasing fit. Row 3 (YIPF5) is noisy at low doses but decreases at higher doses; all methods except ANOVA captured the overall trend without spurious inflections. Row 4 (SPHK1) rises slightly before decreasing; MSstatsResponse remained flat through the rise and transitioned downward only once the data supported a decline.

Similar patterns appeared across other benchmark data sets. In the SRM benchmark (Supplemental Figs. S10–S13), YES, LCK, and CSK show low-dose increases, where MSstatsResponse remains flat until the decline begins. For clearly sigmoidal profiles (e.g., CSK in DIA and TMT), MSstatsResponse closely matched four-parameter log-logistic model fits, recovering sigmoidal shapes when present but not imposing them otherwise. Additional examples for all positive controls are provided in Supplemental Figs. S10–S13.

### In Experimental Data Sets, Statistical Methods Differed in Drug–Target Detection Sensitivity

**Fig. 4:**
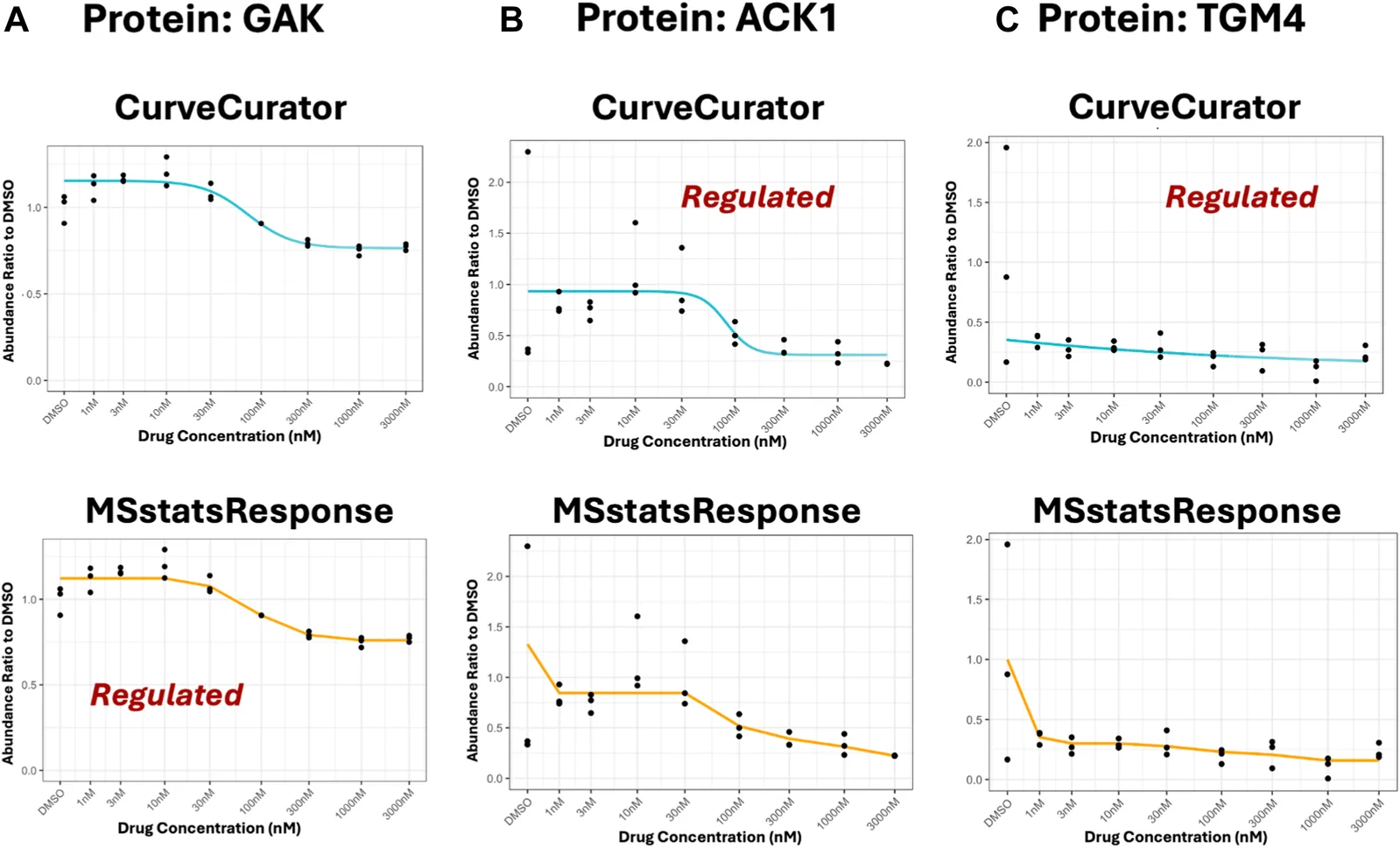
**DIA benchmark: comparison of target identification sensitivity across methods.***A*, MSstatsResponse showed the sensitivity to detect potentially interesting partial responses without overfitting to noise. *B* and *C*, in contrast, sigmoidal methods such as CurveCurator were more sensitive and occasionally detected noisier profiles like ACK1 and TGM4. DIA, data-independent acquisition.

Table 3 summarizes the number of significant proteins detected by each method across acquisitions. Across the different acquisition types, the number of detected interactions varied notably by method. ANOVA recovered all five positive controls across every benchmark, but detected the largest number of significant proteins overall, as high as 90 in the DIA data set, of which only 15 were kinases. This low proportion of kinase hits is consistent with its higher false-positive rate on noninteracting proteins. Among the remaining methods, CurveCurator and MSstatsResponse were the closest, while dr4pl failed to detect all the positive controls except for in the DIA data set. Parametric methods, dr4pl, and CurveCurator, missed ABL1 in the SRM data set (Supplemental Figs. S11 and S12), and MSstatsResponse missed YES in the TMT benchmark with an adjusted *p* value of q = 0.066, but detected it in the TMT-RTS suggesting it was borderline (Supplemental Fig. S10). CurveCurator and MSstatsResponse detected a similar number of kinase hits and largely agreed across acquisitions, though CurveCurator missed GAK, which exhibits a partial response. CurveCurator also picked up additional hits that MSstatsResponse did not, including the kinase TNK2 and a larger number of nonkinase proteins overall (18 versus 3 in DIA), such as TGM4 (Fig. 4).

**Table 3:** Benchmark data set significant protein–drug interactions across acquisition types and methods using all replicates

Method	DIA	SRM	TMT	TMT-RTS
dr4pl	7/9 (55)	4/4 (4)	5/6 (3)	6/7 (3)
CurveCurator	13/31 (5)	6/6 (4)	10/13 (5)	10/11 (5)
ANOVA	15/90 (5)	5/5 (5)	14/21 (5)	12/22 (5)
MSstatsResponse	11/14 (5)	5/5 (5)	9/9 (4)	10/11 (5)

Results using full data sets in DIA, TMT, TMT with RTS, and SRM data sets. Significant hits are reported as [sig. kinases/sig. proteins], where kinases were defined using the UniProt keyword search (KW-0418, *Homo sapiens*, reviewed). Known Dasatinib targets (SRC, ABL1, YES, CSK, and LCK) are shown in parentheses and serve as positive controls.

DIA, data-independent acquisition; RTS, real-time search; SRM, selected reaction monitoring; TMT-DDA, tandem mass tag–based data-dependent acquisition.

### In Pseudo-Single-Replicate Experimental Data Sets, Drug–Target Detection Varied Between Replicates

Single-replicate experimental designs present a challenge for detecting drug–protein interactions using dose–response models. Parametric curve fitting methods are particularly sensitive to variability in the input data. Without replicates, small fluctuations or outliers at a single dose can substantially change the estimated curve shape, resulting in misleading dose–response representations and missed interactions.

**Fig. 5:**
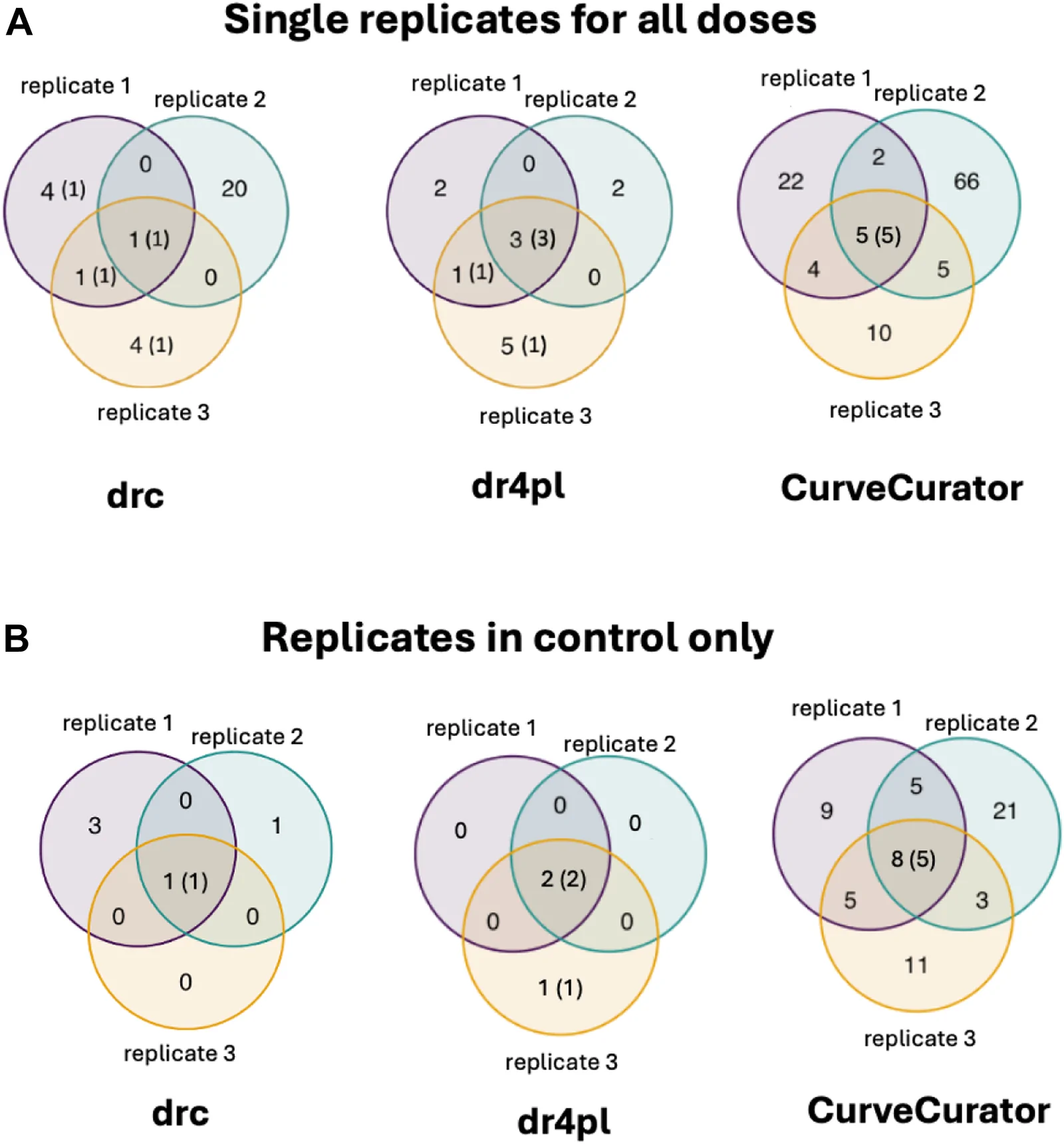
**DIA benchmark: replicate choice influenced detection of drug–protein interactions.***A*, single replicate per dose. *Rows* show drc, dr4pl, and CurveCurator (*top to bottom*). Analyses using only 1 replicate at each dose produced unstable detection: drc showed *low* sensitivity (CSK most consistently detected), dr4pl correctly identified three known Dasatinib targets (SRC, ABL1, and CSK), while only replicate 3 recovered all five targets (SRC, ABL1, CSK, YES, and LCK). CurveCurator recovered all five targets but also detected many additional proteins with substantial between-replicate variability. *B*, replicates in DMSO only. Inclusion of replicates in the control reduced random detections for all methods relative to (*A*). Overall sensitivity remained limited. DIA, data-independent acquisition.

To demonstrate this in practice, we analyzed each replicate from our triple-replicate DIA benchmark experiment independently to mimic three “pseudo” single-replicate design experiments. Figure 5 shows that the results varied greatly depending on which replicate was used, demonstrating the lack of reproducibility in single-replicate experiments. dr4pl and drc failed to pick up all positive controls. Although CurveCurator picked up all the positive controls, the agreement between replicates analyzed varied substantially, with as many as 66 unique proteins detected in the analysis of replicate 2. These patterns were further exaggerated in the other benchmark experiments, where all parametric methods (i.e., dr4pl, drc, and CurveCurator) failed to detect every positive control in each replicate (Supplemental Figs. S14–S16).

To demonstrate the benefit of including replicates in the control condition, we repeated the analysis using all three DMSO replicates. As shown in Figure 5*B*, adding control replicates reduced the number of detected proteins significantly for all replicate groups, indicating that the inclusion of replicates in the DMSO condition can help reduce noise and improve reproducibility. However, overall sensitivity remained limited in methods such as drc and dr4pl without replicates in all doses.

To verify that this poor reproducibility reflects the single-replicate design rather than the quality of the DMSO controls specifically, we followed the standard CurveCurator median absolute deviation-based quality-control procedure and repeated the analysis using the lowest drug dose as an alternative reference control (details in Supplemental Sec. 4.5.1). In both analyses the poor reproducibility remained, pointing to design rather than to DMSO sample quality.

### In Simulated Data Sets, MSstatsResponse Detected Partial Responses Without Increasing False Positives

All statistical methods effectively detect proteins with complete responses under ideal experimental conditions (i.e., with low variability, a high number of doses, and multiple replicates per dose). The partial-response category was included to test whether a method can detect incomplete monotonic signal without inflating false positives, independent of why a given curve is incomplete or low in fold-change. For the more difficult case of detecting partial responses, the semiparametric approaches, MSstatsResponse and ANOVA, showed increased sensitivity while maintaining control over false positives.

**Fig. 6:**
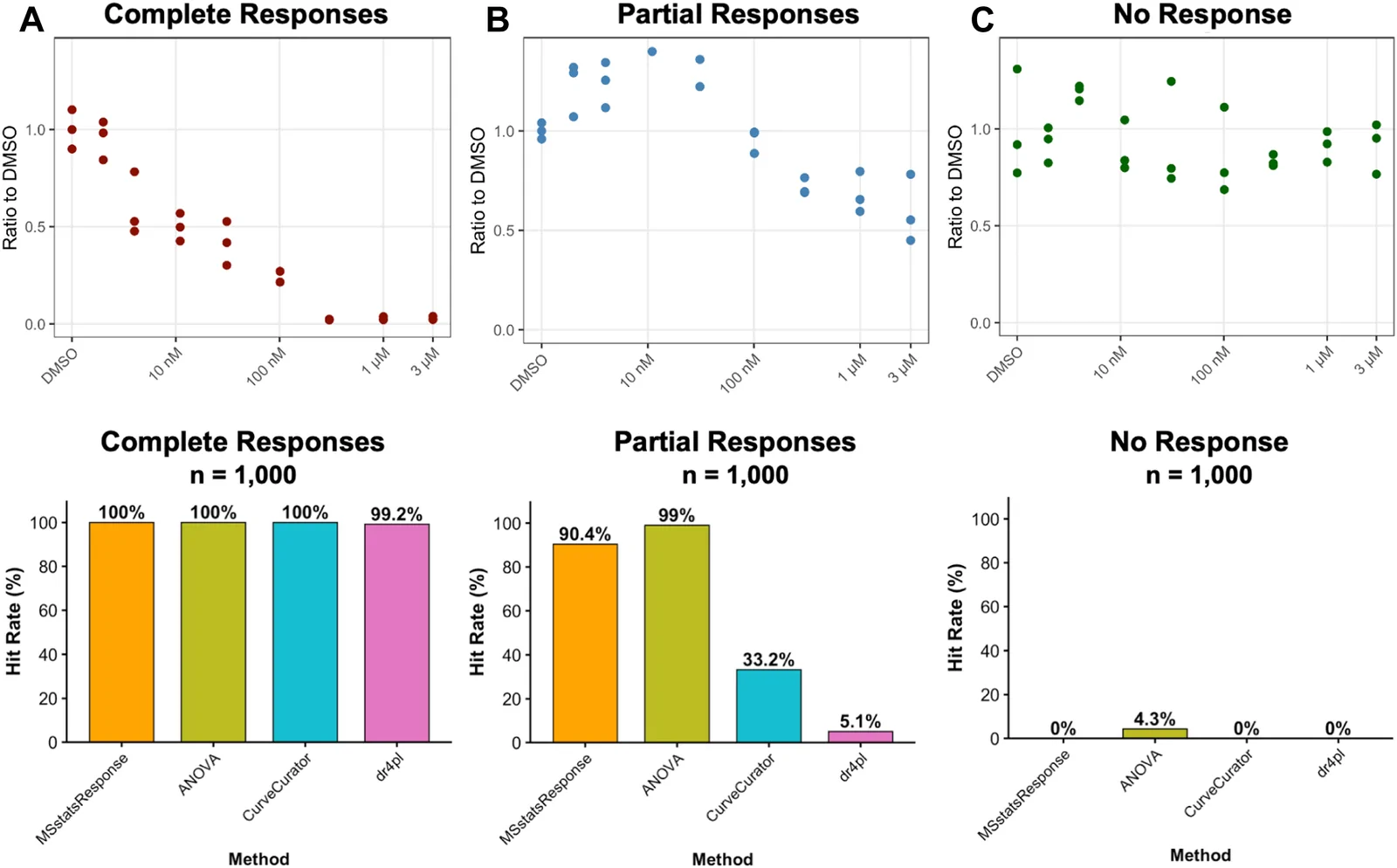
**Simulated data set: semiparametric methods, ANOVA and MSstatsResponse, achieved best target identification of proteins with partial responses.***A*–*C*, simulated protein response profile examples based on real DIA-derived templates, representing a complete, partial, or no response to drug treatment. *A*, a complete-response protein simulated using the SRC response profile. *B*, a partial-response protein simulated using the TEC response profile. *C*, a noninteracting protein simulated using the EIF2AK4 profile. Each bar chart shows the hit rate across 1000 simulated proteins per response category (3000 total), based on 9 doses (DMSO, 1 nM, 3 nM, 10 nM, 30 nM, 100 nM, 300 nM, 1000 nM, 3000 nM), 3 replicates per dose, and a variance of 0.4. All methods perform well under ideal conditions. MSstatsResponse and ANOVA demonstrate high sensitivity in detecting both complete and partial responses, while MSstatsResponse does so without compromising specificity (i.e., no false positives). DIA, data-independent acquisition.

Figure 6 demonstrates this in practice by evaluating each method’s performance on simulated complete, partial, and noninteracting protein profiles with a triple-replicate, 9-dose experimental design derived from the experimental DIA data set. Semiparametric methods, MSstatsResponse and ANOVA, both achieved high sensitivity, detecting all proteins with complete responses (100%) and the majority of partial responses (90.4% and 99%, respectively). However, only MSstatsResponse did so without false positives on noninteracting proteins (0%), whereas ANOVA produced false positives (4.3%), making MSstatsResponse the only method to combine high sensitivity with full specificity. CurveCurator’s lower sensitivity for partial responses is consistent with its fold-change filter (fc = 0.5, held constant throughout; see Evaluation Strategy), which screens out smaller responses to help control false positives while retaining larger ones.

When data variability increased, all methods declined in sensitivity for detecting proteins with partial responses (Supplemental Fig. S19). Notably, the performance of dr4pl dropped substantially under noisy conditions, and failed to detect almost all proteins with complete responses. This highlights the robustness of semiparametric methods, and the dependence of parametric methods on high data quality.

### In Simulated Data Sets with Fewer Doses, MSstatsResponse Maintained Drug–Target Detection Sensitivity

Experimental designs with fewer doses are sometimes necessary in chemoproteomics due to cost and sample space limitations. To evaluate method performance under such constraints, we simulated a data set with four dose conditions (DMSO, 1 nM, 1000 nM, 3000 nM), each with three replicates, across 1000 proteins per response category (3000 total) with a variance of 0.4.

**Fig. 7:**
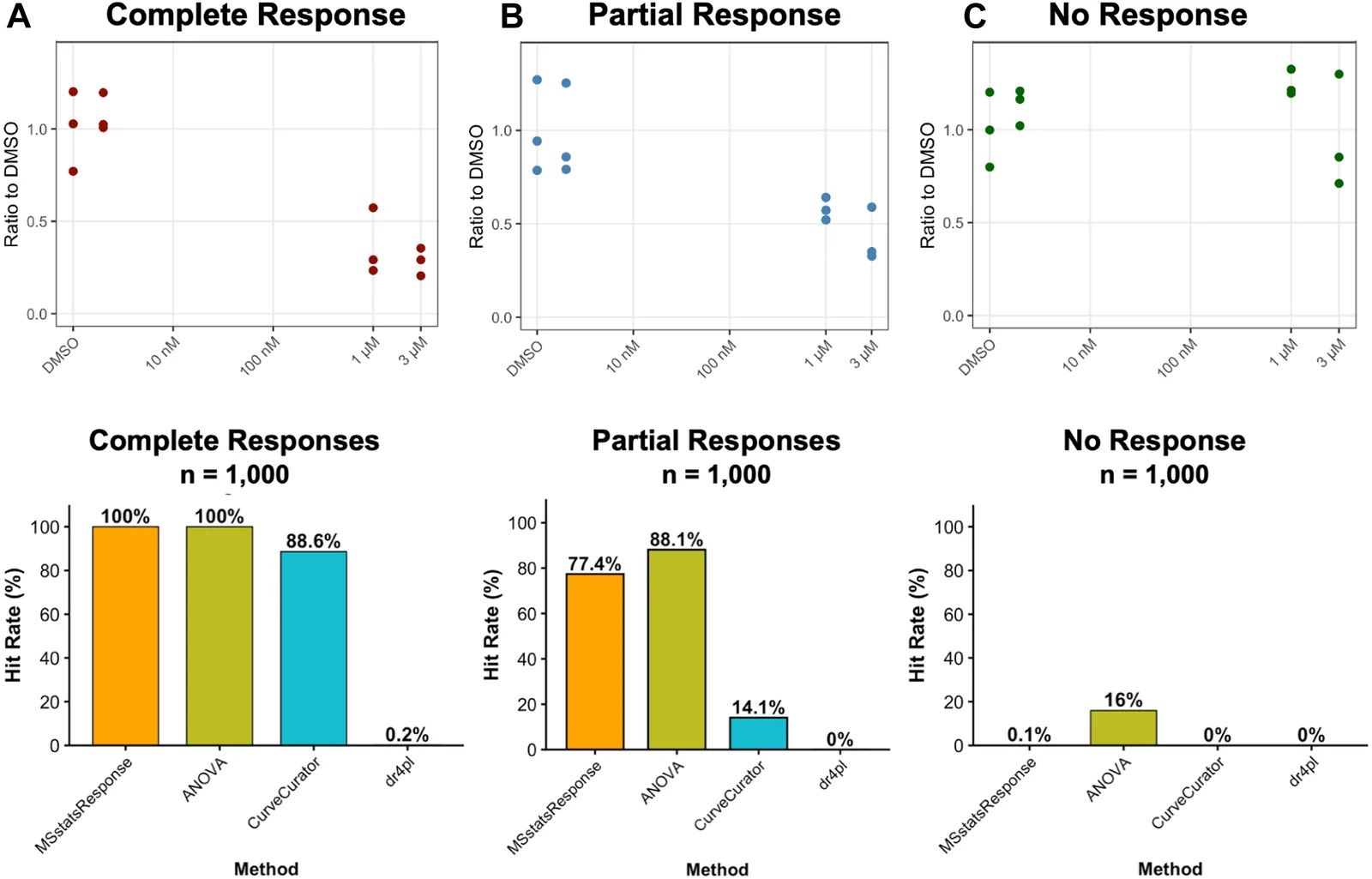
**Simulated data set: MSstatsResponse maintained high sensitivity for detecting proteins with complete responses without increased false-positive rate for data sets with fewer doses.***A*–*C*, simulated protein response profile examples based on real DIA-derived templates, representing a complete, partial, or no response to drug treatment. Method performance evaluated on a simulated data set with 4 doses (DMSO, 1 nM, 1000 nM, 3000 nM), 3 replicates per dose, and a variance of 0.4 across 1000 proteins per response category (3000 total). *A* and *B*, MSstatsResponse and ANOVA maintain high sensitivity despite fewer dose points. CurveCurator retains moderate sensitivity but begins to lose power under reduced dose designs. dr4pl is unable to operate on data sets with only four dose levels due to model fitting constraints. *C*, specificity analysis for noninteracting proteins showed that ANOVA is sensitive to dose condition outliers, leading to false positives. In contrast, MSstatsResponse retains both high sensitivity and specificity by the model’s monotonicity constraint. DIA, data-independent acquisition.

Figure 7 shows that MSstatsResponse and ANOVA maintained high sensitivity for proteins with complete responses despite reduced dosage coverage. CurveCurator showed moderate sensitivity but began to lose power under these conditions, while dr4pl failed to detect proteins with complete responses entirely. These results demonstrate that classic parametric curve fitting methods require many doses to perform effectively. MSstatsResponse, by contrast, effectively controlled false positives through its monotonicity constraint, whereas ANOVA was more sensitive to outliers, leading to inflated false-positive rates among noninteracting proteins. Beyond the number of doses, dose spacing also influences performance. For target identification, prioritizing low and high concentrations is most effective, while sigmoid-based methods are more sensitive to spacing choices given their reliance on capturing the full response shape.

**Fig. 8:**
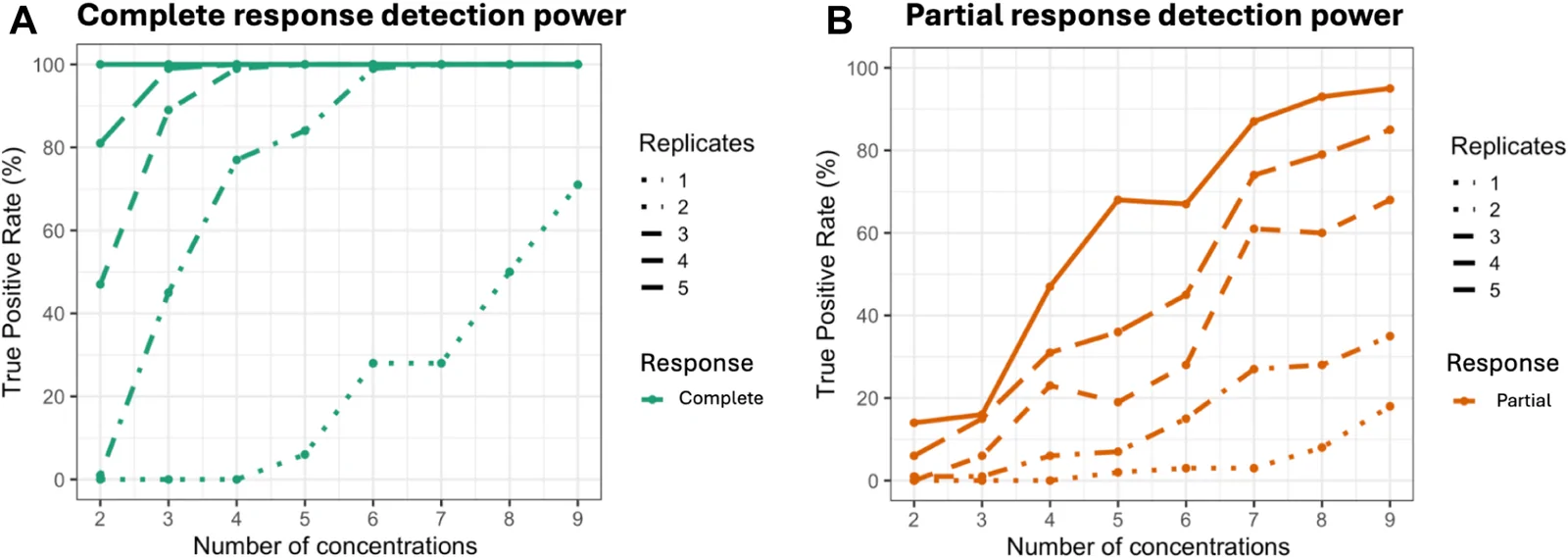
**Simulated data set: detected protein responses depended on the number of doses and replicates per dose.** Simulated dose–response experiments were generated using increasing numbers of concentrations (in nM): 2-dose (0, 3000), 3-dose (0, 1000, 3000), 4-dose (0, 1, 1000, 3000), 5-dose (0, 1, 100, 1000, 3000), 6-dose (0, 1, 100, 300, 1000, 3000), 7-dose (0, 1, 10, 100, 300, 1000, 3000), 8-dose (0, 1, 10, 30, 100, 300, 1000, 3000), and 9-dose (0, 1, 3, 10, 30, 100, 300, 1000, 3000). *A*, detection of proteins with complete responses (i.e., proteins whose probe-bound abundance decreases to near zero at the highest dose) approached 100% with 5 replicates and as few as 2 doses. Experiments with 6+ doses required at least two replicates for reliable detection. *B*, detection of proteins with partial responses (i.e., proteins whose probe-bound abundance decreases to roughly 50% of the control level) required more replicates and more doses. In experimental data sets, MSstatsResponse produced precise OC50 estimates.

To generalize beyond a single simulated design, we generated power curves across a range of scenarios (Fig. 8). These curves show that complete responses can be detected with relatively few doses, provided replicates are included, while partial responses require both increased dose levels and replication for reliable detection. To assess whether these trends extend to experimental data, we applied the same analysis framework to benchmark data sets with fewer dose levels. In this setting, target detection patterns were broadly comparable across acquisition types (Supplemental Table S5). For experiments involving a single high-dose treatment, we recommend standard differential abundance methods (e.g., MSstats (40)), which are better suited for single-dose target identification. Overall, these results demonstrate that semiparametric approaches like MSstatsResponse can maintain robust performance with fewer doses, offering a practical alternative design strategy when resources are limited.

### In experimental datasets, MSstatsResponse produced precise OC50 estmates

Among the methods compared, MSstatsResponse and dr4pl provide CIs for OC50 estimation, whereas CurveCurator produces only a single point estimate and ANOVA does not support this parameter at all. We therefore focused our evaluation on MSstatsResponse and dr4pl, and included CurveCurator’s point estimates for reference.

We compared these methods across three experimental design scenarios (all with three replicates per dose): a full 9-dose design (DMSO + 8 doses), a reduced 5-dose design (2 low, 1 middle, 2 high), and minimal 4-dose design (2 low, 2 high).

**Fig. 9:**
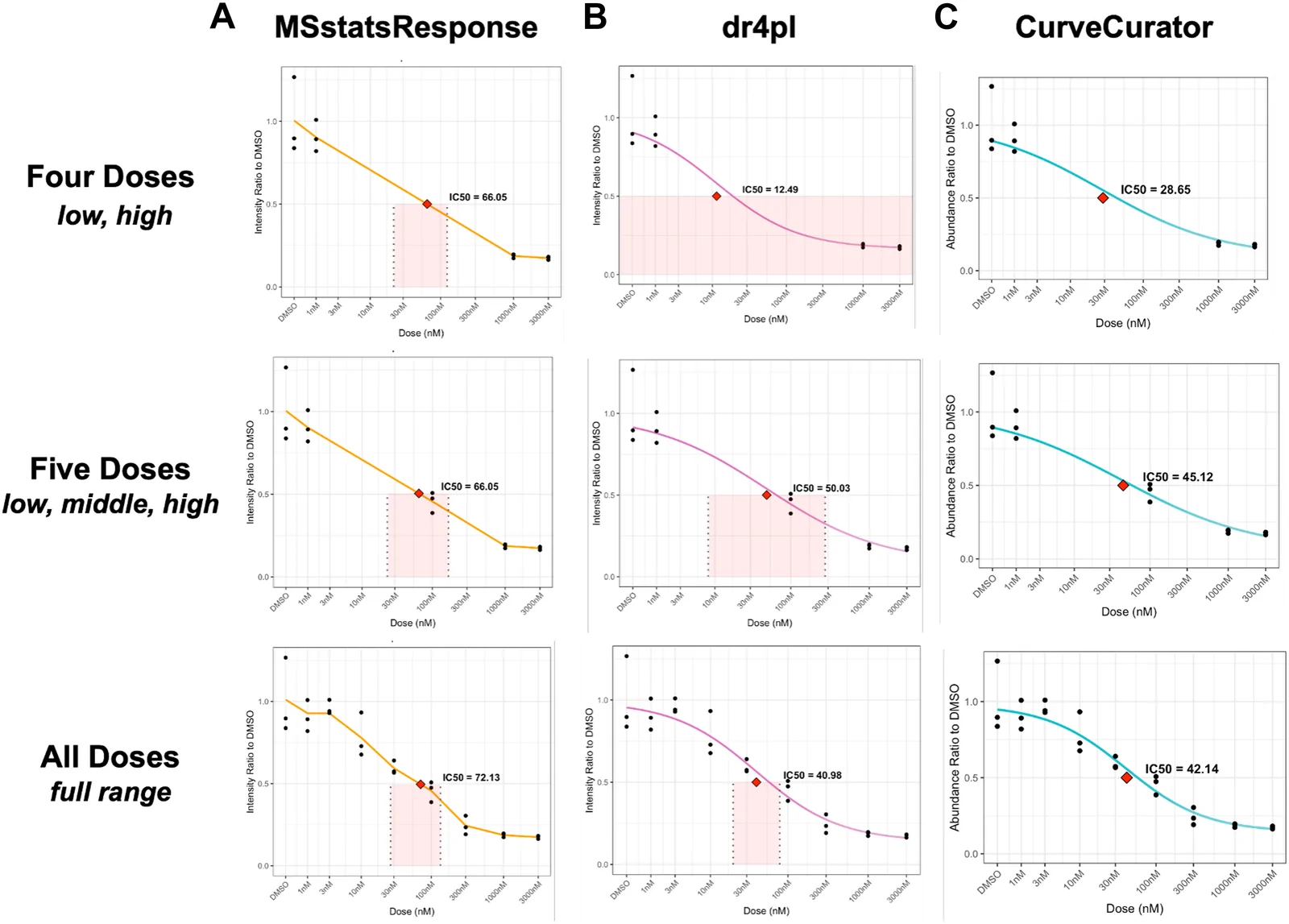
**DIA benchmark: MSstatsResponse maintained stable OC50 estimates across experimental designs with fewer doses.** Columns compare three estimation methods, MSstatsResponse, dr4pl, and CurveCurator, across varying experimental designs. *A*, MSstatsResponse produced consistent OC50 estimates with narrow bootstrapped confidence intervals across four-, five-, and nine-dose designs. *B*, dr4pl required more dose points to achieve stability. Without a middle dose, confidence intervals were wide or unrealistic. *C*, CurveCurator generated stable single OC50 point estimates in all designs. No confidence intervals are provided to assess uncertainty. DIA, data-independent acquisition.

Figure 9 shows OC50 estimates and 95% CIs for the drug target ABL1 under each design. While both methods yielded similar results with the full-dose experiment, dr4pl produced increasingly wide and unstable CIs as doses were removed. This instability stems from dr4pl′s reliance on a sigmoidal dose-response assumption, which cannot be reliably fit with limited dose points. CurveCurator generated a single OC50 estimate in each scenario, but without CIs its reproducibility and statistical interpretability were limited.

In contrast, MSstatsResponse combined isotonic regression with linear interpolation and bootstrapping to generate stable and reproducible OC50 estimates with valid CIs under all scenarios. Because it assumes only that dose–response relationships are monotonic (nonincreasing), rather than strictly sigmoidal, MSstatsResponse remained robust even when the number of available doses was limited. These results highlight its advantage in experimental settings where cost or sample constraints restrict dose coverage. Despite these results, we still recommend including as many dose levels as feasible when the experimental goal is precise and reliable OC50 estimation.

## Discussion

We propose MSstatsResponse, a semiparametric statistical framework for the analysis of chemoproteomics experiments. Our results demonstrate that MSstatsResponse is well positioned to analyze large-scale proteomics drug screens across a range of experimental designs and acquisition strategies. Unlike fully parametric models, which fit curves of prespecified shape and required many doses and replicates to achieve stability, MSstatsResponse was able to maintain sensitivity in more resource-limited settings.

A key finding of this study is that experimental design choice strongly influenced the reliability of dose–response analysis results. While large-scale screens often favor single-replicate, multidose designs, and some tools recommend prioritizing dose coverage over replication (68), we showed that these designs were highly sensitive to experimental noise and exhibited poor reproducibility (Fig. 5). In these cases, parametric models frequently overfit or misinterpreted random fluctuations which lead to increased false positives. In contrast, a minimal dose, multi-replicate design provided a more reliable foundation for initial hit detection. We therefore recommend this design as a starting point for exploratory screens, followed by targeted dose–response profiling to assess potency.

We recommend at the very least, for exploratory screens to include replicates in the control condition. Without replicated controls, ratio-scale normalization can amplify unseen variability in the control condition which can artificially distort downstream dose–response measurements. We show in Figure 5 that the inclusion of replicates in the control condition can at the very least control the false-positive rate and limit sensitivity to random fluctuations in the data set.

For confirmatory analyses, adding multiple doses with replication provided the strongest foundation for both interaction detection and OC50 estimation. As demonstrated in our simulations and benchmarks, this design enabled more accurate curve fitting, increased sensitivity to proteins with a partial response to drug treatment, and more stable parameter estimates (Figs. 6 and 9). Although this option is more resource-intensive, this design is strongly recommended when the analysis goal is high-confidence detection and potency estimation.

We recognize that reducing the number of drug doses is sometimes necessary due to cost limitations. We found that MSstatsResponse retained robust performance with as few as four doses with replicates (Fig. 7). In contrast, fully parametric models such as dr4pl often failed under reduced designs, as the limited dose range was insufficient to fit all curve parameters reliably.

We further showed that all acquisition strategies tested (i.e., DIA, DDA, and SRM) were suitable for chemoproteomics dose–response analysis (Table 3). We conclude that the acquisition method selection can be guided primarily by instrument availability, throughput requirement, and the overall practitioner’s expertise.

Altogether, our findings lead to three major recommendations. First, when resources are limited, we recommend prioritizing biological replicates while limiting the number of doses. Replicates consistently improved robustness across all methods and tasks and were essential for reliable inference. Second, when possible, combine replication with at least 1 intermediate and 1 high dose to enable flexible curve modeling and accurate OC50 estimation. MSstatsResponse is particularly well suited to these designs, as it provided robust inference without relying on strict curve assumptions. And third, if deciding to do a single-replicate dose–response screen, we strongly recommend the inclusion of replicates in the control condition to stabilize baseline estimates, reduce false positives, and further increase screen reproducibility.

Beyond chemoproteomics, the MSstatsResponse framework is readily extendable to a wide range of dose–response–like studies. These include transcriptomics dose–response experiments, thermal profiling experiments where temperature replaces dose, protein turnover studies, or more generally, any biological relationship that follows a monotonic trend. In this study, we focused on non-increasing constraints, reflecting the behavior of inhibitors, but MSstatsResponse can be applied equally well to data sets where responses are expected to be nondecreasing. The underlying methods and workflow remain unchanged. MSstatsResponse is also available through the MSstatsShiny online GUI which streamlines data processing, analysis, and visualization for users. Future work will focus on formalizing dedicated workflows and vignettes for additional data modalities.

## Data Availability

The four benchmark data sets analyzed in this article are publicly available on MassIVE under the accession numbers MSV000100970 (DIA dose–response data set), MSV000100968 (TMT dose–response data sets), and MSV000100969 (SRM dose–response data set). The intermediate data sets and analysis scripts used to reproduce the results in this article are available on Zenodo at https://doi.org/10.5281/zenodo.18881723. The MSstatsResponse R package is available through Bioconductor at https://bioconductor.org/packages/MSstatsResponse. The MSstatsShiny GUI tool is also available through Bioconductor at https://bioconductor.org/packages/MSstatsShiny.

## Supplemental Data

This article contains supplemental data (21).

## Conflict of Interest

The authors declare no competing interests.
