# Supplementary material for: MSstatsResponse: Semiparametric Statistical Model Enhances Detection of Drug–Protein Interactions in Chemoproteomics Experiments
Source: Mol Cell Proteomics. 2026 Aug 12;25(9):101637. doi: 10.1016/j.mcpro.2026.101637 (PMC13580065; doi:10.1016/j.mcpro.2026.101637)
Supplement: Supplementary file 1 [file mmc1.pdf]

MSstatsResponse: Semi-parametric statistical model enhances detection of drug-protein interactions in chemoproteomics experiments

Supplementary Information

Sarah Szvetecz[1,2] Devon Kohler[1,2] Joel D. Federspiel[3] S. Denise Field[4]
Pierre Jean-Beltran[4] Robert J. Seward[3] Hyunsuk Suh[3] Liang Xue[4]
Olga Vitek[1,2*]

[1]Khoury College of Computer Sciences, Northeastern University, Boston, MA, USA

[2]Barnett Institute for Chemical and Biological Analysis, Northeastern University, Boston, MA, USA 02115

[3]Pfizer Inc., Andover, MA, USA

[4]Pfizer Inc., Cambridge, MA, USA

*Corresponding author: o.vitek@northeastern.edu

Contents

1 Experimental datasets **3**

1.1 Benchmark experiments . . . 3

1.2 Simulated datasets . . . 5

2 Background **7**

3 Results **10**

3.1 Overall workflow in MSstatsResponse . . . 10

4 Evaluation **13**

4.1 Evaluation strategy . . . 13

4.2 All acquisition strategies were suitable for chemoproteomics experiments . . . 14

4.3 In experimental datasets, statistical methods produced distinct dose-response curves . . . 15

4.4 In experimental datasets, statistical methods differed in drug-target detection sensitivity . . . 18

4.5 In pseudo-single-replicate experimental datasets, drug-target detection varied between replicates 19

4.5.1 CurveCurator MAD-based quality control analysis . . . 20

4.6 In simulated datasets, MSstatsResponse detected partial responses without increasing false positives . . . 23

4.7 In simulated datasets with fewer doses, MSstatsResponse maintained drug-target detection sensitivity . . . 24

1 Experimental datasets

1.1 Benchmark experiments

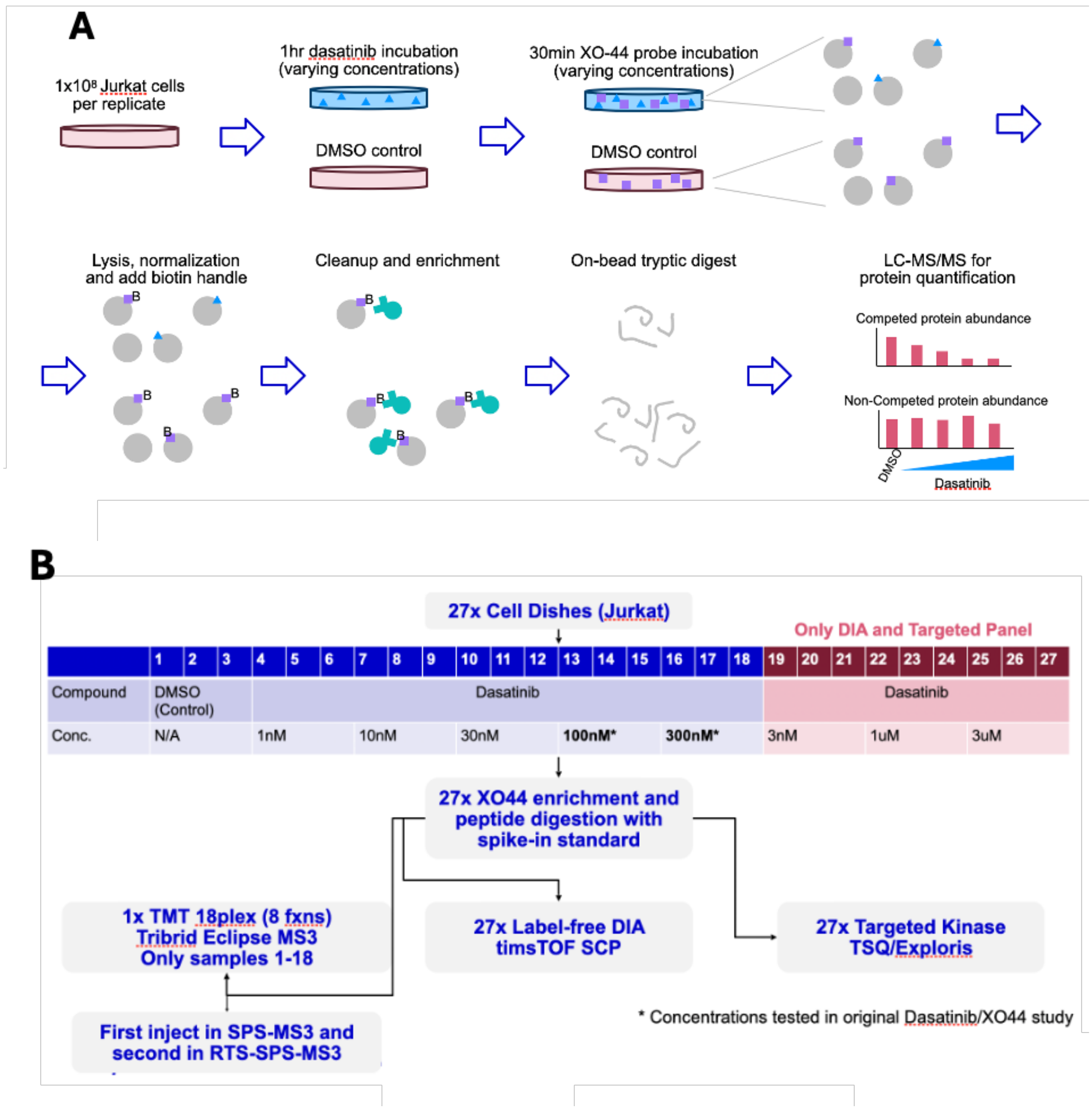

Figure 1: **Benchmark datasets: experimental design. (A)** Cells were treated with varying doses of Dasatinib in competition with the XO44 pan-kinase probe and analyzed using data-independent acquisition (label-free DIA), data-dependent acquisition (TMT 18-plex, standard MS3 and real-time search), and targeted selected reaction monitoring (SRM). **(B)** All datasets included triplicate biological replicates per condition. TMT datasets included five drug doses (1 nM, 10 nM, 30 nM, 100 nM, and 300 nM) plus DMSO controls. DIA and SRM datasets included eight drug doses (1 nM, 3 nM, 10 nM, 30 nM, 100 nM, 300 nM, 1 μM, and 3 μM) plus DMSO controls.

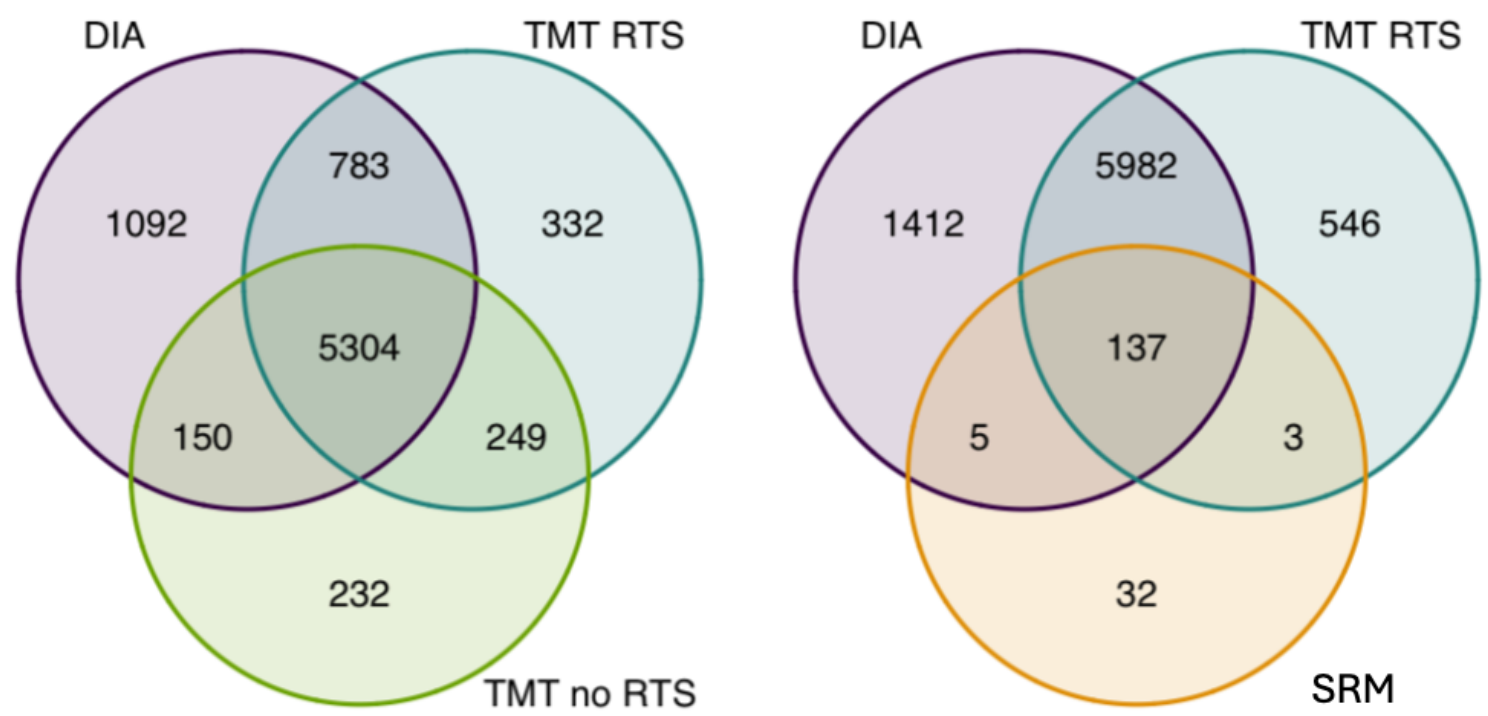

Figure 2: **Benchmark datasets: number of proteins identied per acquisition.** All datasets successfully identified the five known Dasatinib targets (SRC, ABL1, YES, LCK, and CSK) and the negative control EIF2AK4. Real-time search improved peptide identification in the TMT workflow, increasing protein coverage by 733 proteins relative to standard MS3 acquisition.

1.2 Simulated datasets

The simulations of log-scale protein abundances used as a template the abundances of a selected protein from a past dose-response experiment. In this manuscript, the template proteins were chosen from the DIA benchmark dataset to represent complete, partial, or no responses. The corresponding template abundances were derived by averaging the log2-transformed protein intensities across replicates at each dose level.

Additional variation was added at each dose level, to introduce randomness and create the number of desired replicates. In this manuscript, the variation was estimated from the average protein-level variance across sample groups in the DIA benchmark dataset.

Outliers were optionally added to some simulations by adding values from the log-normal distribution. In this manuscript, the probability of an outlier was set to 0 as default, and set to 5% when investigating outliers.

Algorithm 1 Simulation of dose–response Data

Input:
D: number of drug doses (excluding control)
R: number of replicates per dose
σ^2: technical and biological variance
$\{\mu_d, d = 0, \ldots, D\}$: template protein abundances (e.g., complete, partial, no response) per dose
p_{outlier}: probability of an outlier

Output:
$\{y_{dr}, d = 0, \ldots, D;\ r = 1, \ldots, R\}$: simulated protein-level log-abundances across doses and replicates

for $d = 0$ to D **do**
  for $r = 1$ to R **do**
    $y_{dr} \leftarrow \mu_d + \mathcal{N}(0, \sigma^2)$ ▷ Add noise to template value

    $isMissing \leftarrow Bernoulli(p_{\text{outlier}})$ ▷ Random outlier selection
    if $isMissing = 1$ **then**
      $s \leftarrow 2 \cdot Bernoulli(0.5) - 1$ ▷ Random direction selection
      $y_{dr} \leftarrow y_{dr} + s \cdot \text{LogNormal}(0, 1)$ ▷ Apply log-Normal deviation
    end if
  end for
end for
Output: $\{y_{dr}, d = 0, \ldots, D;\ r = 1, \ldots, R\}$

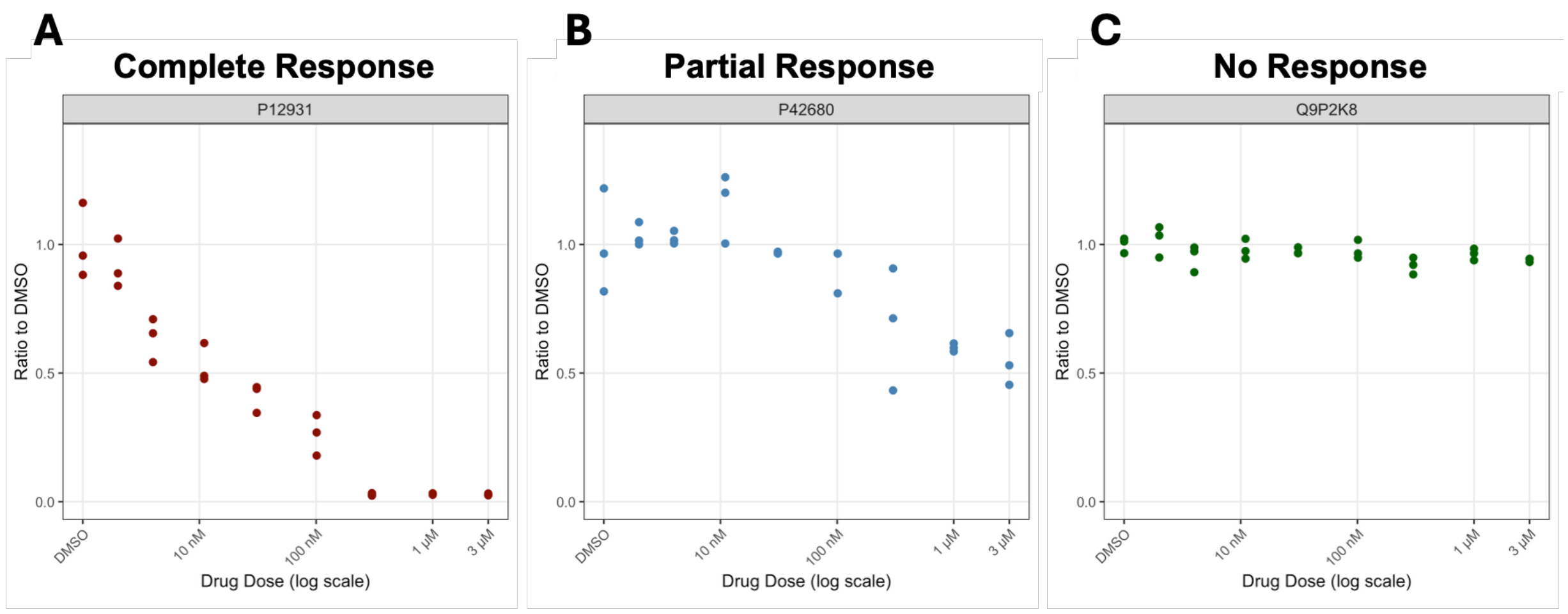

Figure 3: **DIA benchmark: protein templates chosen for simulation. (A)** SRC protein, complete response. Complete-response templates represented protein abundance decreasing to near zero at the highest drug dose. **(B)** TEC protein, partial response. Partial-response templates represented protein abundance decreasing to approximately 50% of baseline levels. **(C)** EIF2AK4 protein, no response. Non-interacting proteins maintained constant abundance across all doses, indicating no drug effect.

2 Background

Model	Hypothesis testing	Reject H_0	Degrees of freedom	OC50
**drc**	$H_0 : \theta_3 = 0 \quad \text{or} \quad \theta_2 \notin [x_{\min}, x_{\max}]$<br>$H_a : y_{dr} = \theta_1 + \frac{\theta_4 - \theta_1}{1 + 10^{\theta_3(x_d - \log_{10}\theta_2)}} + \epsilon_{dr}$<br>$\epsilon_{dr} \overset{\text{iid}}{\sim} N(0, \sigma^2)$	$\hat{\theta}_3 < 0$ and<br>$\hat{\theta}_2 \in [x_{\min}, x_{\max}]$ and<br>p-value for θ_3 and $\theta_2 < \alpha$	N/A	θ_2
**dr4pl**	$H_0 : \theta_3 = 0 \quad \text{or} \quad \theta_2 \notin [x_{\min}, x_{\max}]$<br>$H_a : y_{dr} = \theta_1 + \frac{\theta_4 - \theta_1}{1 + 10^{\theta_3(x_d - \log_{10}\theta_2)}} + \epsilon_{dr}$<br>$\epsilon_{dr} \overset{\text{iid}}{\sim} N(0, \sigma^2)$	$\hat{\theta}_3 < 0$ and<br>$\hat{\theta}_2 \in [x_{\min}, x_{\max}]$ and<br>95% CI for θ_2 excludes 0	N/A	θ_2
**CurveCurator**	$H_0 : y_{dr} = \mu + \epsilon_{dr}$<br>$H_a : y_{dr} = \theta_1 + \frac{\theta_4 - \theta_1}{1 + 10^{\theta_3(x_d - \log_{10}\theta_2)}} + \epsilon_{dr}$<br>$\epsilon_{dr} \overset{\text{iid}}{\sim} N(0, \sigma^2)$	$F_{obs} = \frac{\text{SSE}_0 - \text{SSE}_a}{\text{SSE}_a} \cdot \frac{DR}{4}$<br>$> F(1-\alpha, df_1, df_2)$<br>and $\lvert \hat{y}'_{x_{\min}} - \hat{y}'_{x_{\max}} \rvert > \delta$	$df_1 = 5$<br>$df_2 = (0.8 - c) \times$<br>$(DR - 2.5)$, where<br>$c = \frac{1}{\frac{(DR-4)^4}{DR} + 4}$	θ_2
**ANOVA**	$H_0 : y'_{dr} = \mu + \epsilon_{dr}$<br>$H_a : y'_{dr} = \mu + x_d + \epsilon_{dr}$<br>$\sum_{d=0}^{D} x_d = 0, \quad \epsilon_{dr} \overset{\text{iid}}{\sim} N(0, \sigma^2)$	$F_{obs} = \frac{\frac{\text{SSE}_0 - \text{SSE}_a}{df_0 - df_a}}{\frac{\text{SSE}_a}{df_a}}$<br>$> F(1-\alpha, df_0 - df_a, df_a)$	$df_0 = (D+1)R - 1$<br>$df_a = (D+1)(R-1)$	N/A
**MSstatsResponse**	$H_0 : y'_{dr} = \mu + \epsilon_{dr}$<br>$H_a : y'_{dr} = f(x_d) + \epsilon_{dr}$<br>$\epsilon_{dr} \overset{\text{iid}}{\sim} N(0, \sigma^2)$, where<br>$f(x_0) > f(x_1) > \cdots > f(x_D)$	$F_{obs} = \frac{\frac{\text{SSE}_0 - \text{SSE}_a}{df_0 - df_a}}{\frac{\text{SSE}_a}{df_a}}$<br>$> F(1-\alpha, df_0 - df_a, df_a)$	$df_0 = (D+1)R - 1$<br>$df_a = (D+1)R - y^*$	N/A
	$H_0 : y_{dr} = \mu + \epsilon_{dr}$<br>$H_a : y_{dr} = f(x_d) + \epsilon_{dr}$<br>$\epsilon_{dr} \overset{\text{iid}}{\sim} N(0, \sigma^2)$, where<br>$f(x_0) > f(x_1) > \cdots > f(x_D)$	N/A	N/A	$x_d : f(x_d) = 0.5$

Table 1: **Summary of statistical modeling and inference for one protein, used for task 2 (target identification at level α), and task 3 (OC50 estimation).** Notation is as in the main text. y_{dr} are protein abundances on the ratioed scale; y'_{dr} are protein abundances on $\log_2$-transformed (or compatible) scale. D is the number of doses excluding the control. The table assumes an equal number of replicates R for each dose. SSE_0 and SSE_a are the sum of squares of the error of the null and alternative models, respectively. F is the probability distribution. $\hat{}$ refers to values estimated from the data. y^* is the number of unique fitted values used to fit the isotonic regression model. In proteins where the monotonicity constraints are not broken, $y^* = D + 1$. For convenience of interpretation, MSstatsResposne fits the isotonic regression on the $\log_2$-transformed scale for task 2, and on the ratioed scale for task 3. However, OC50 can be equivalently estimated from the log-scale fit as $\log_2\left(\frac{1}{2}\hat{y}_{0\cdot})\right) = \hat{f}(x_0) - 1$.

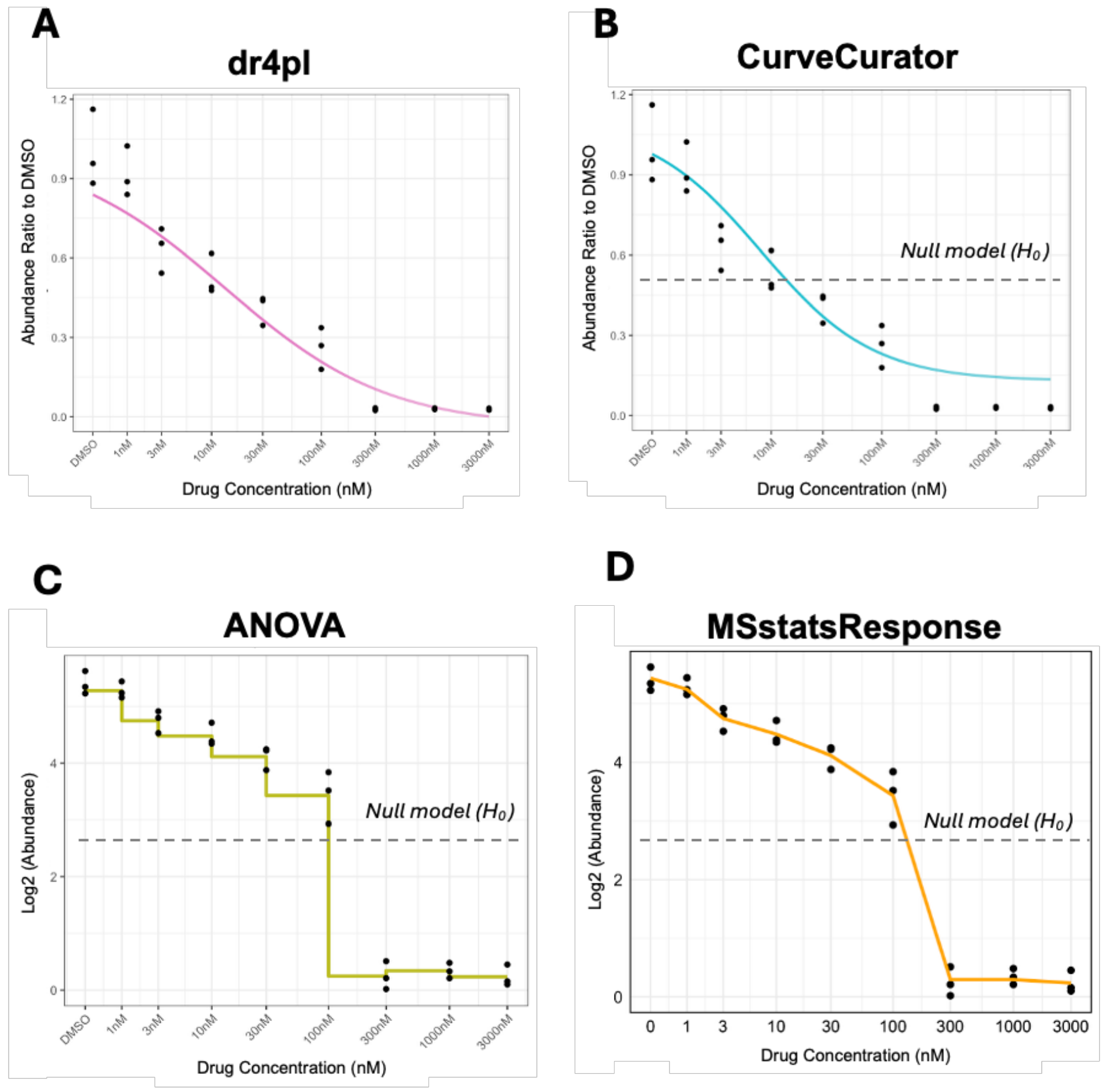

Figure 4: **DIA benchmark, positive control protein SRC.** Dose-response curves for SRC, a known Dasatinib target, are shown for two parametric methods(**(A)** dr4pl, **(B)** CurveCurator) and two semi-parametric (**(C)** ANOVA, **(D)** MSstatsResponse). Each panel displays the fitted model curve and the null hypothesis used for target identification.

3 Results

3.1 Overall workflow in MSstatsResponse

Task 1: Curve fitting

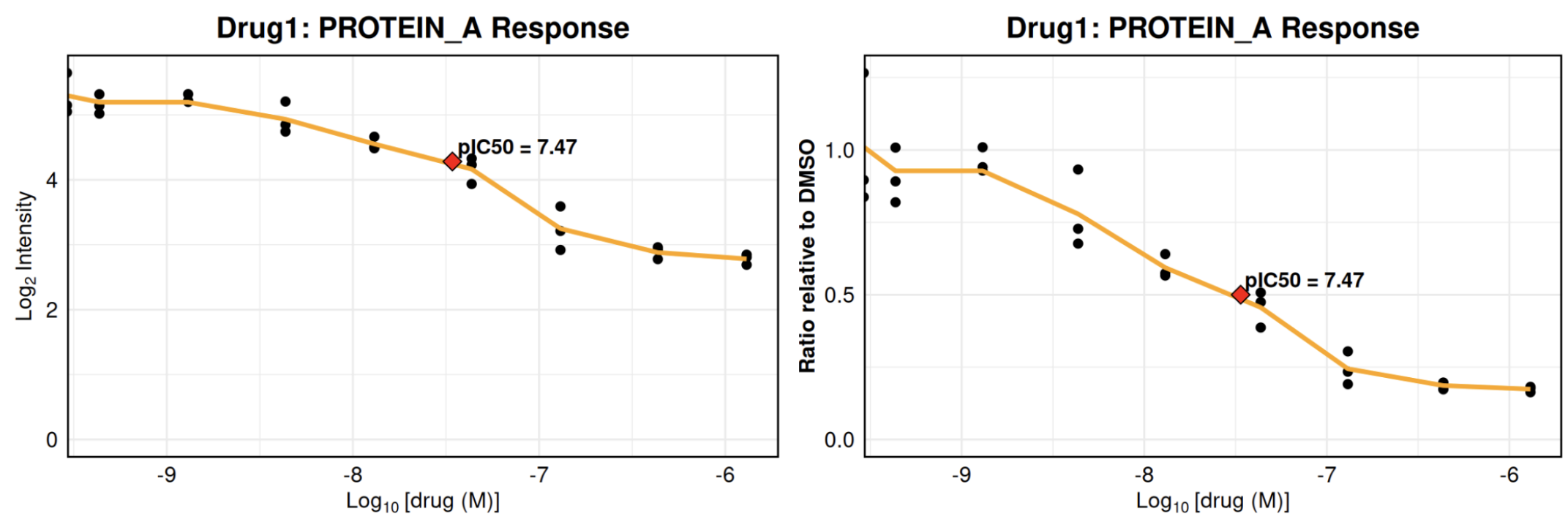

Figure 5: **Simulated protein: dose-response curves on the $\log_2$ and ratio scale for the same protein.** The same simulated protein is fit with MSstatsResponse on the $\log_2$-intensity scale (left) and the ratio-to-DMSO scale (right). Because the log transformation is monotonic, the two fits are equivalent and yield the same OC50 estimate. Users can visualize fitted curves on either scale.

Task 2: Target identification

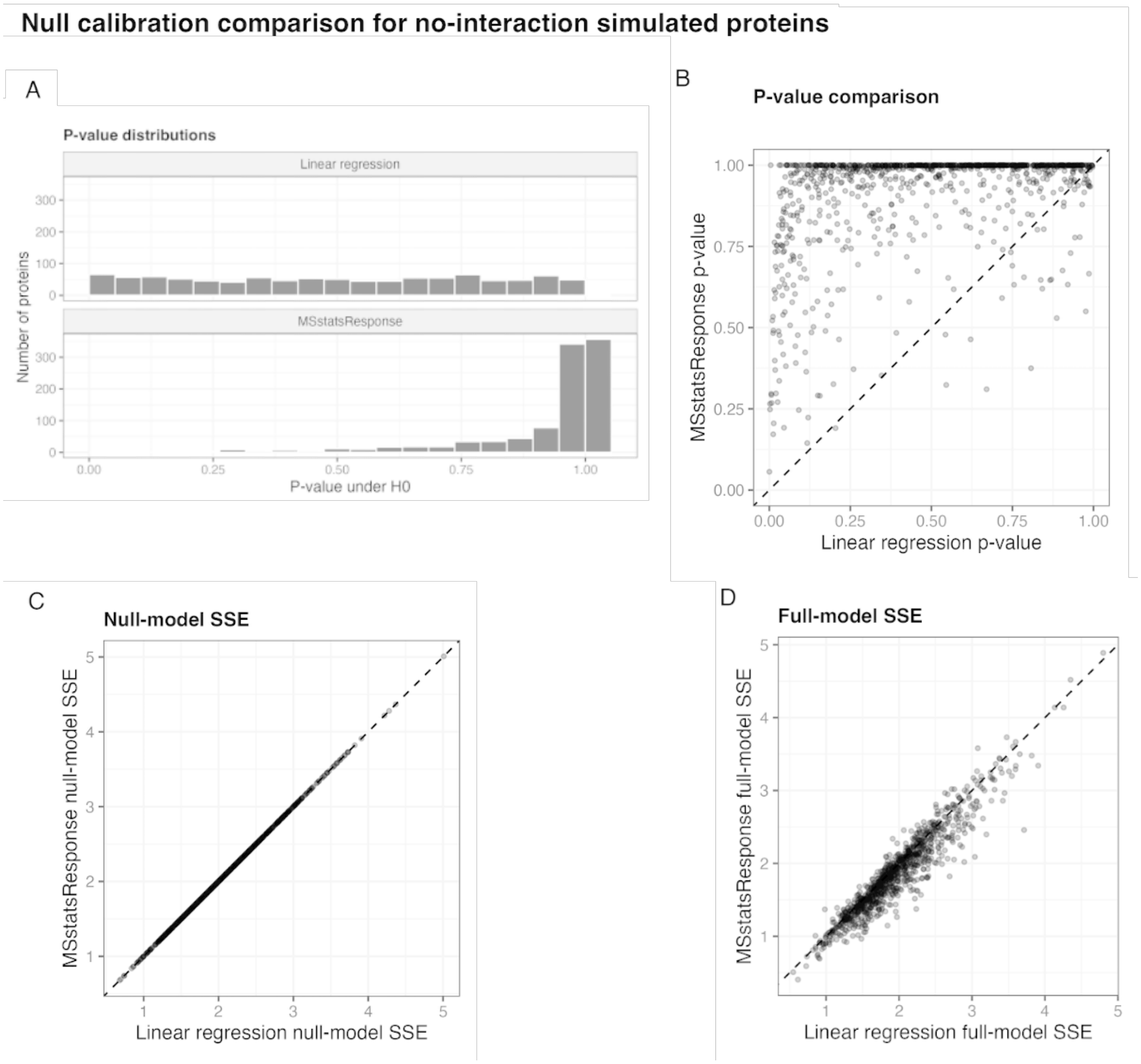

Figure 6: **Null calibration of the MSstatsResponse isotonic F-test compared to linear regression.** Both tests were applied to the same simulated no-response (null) proteins. **(A)** P-value distributions under the null: linear regression p-values are approximately uniform, while MSstatsResponse p-values are shifted toward 1 for the same proteins. **(B)** Direct comparison of p-values for each protein; MSstatsResponse p-values are systematically larger than the corresponding linear regression p-values (points above the diagonal). **(C)** Null-model residual sum of squares (SSE) is identical between the two approaches, as both fit a flat model to the same data. **(D)** Full-model SSE is comparable between approaches, indicating similar residual fit. Together, panels (C) and (D) show that the conservative p-values do not arise from a difference in model fit, but from how the reduction in SSE is scaled by degrees of freedom at the inference step

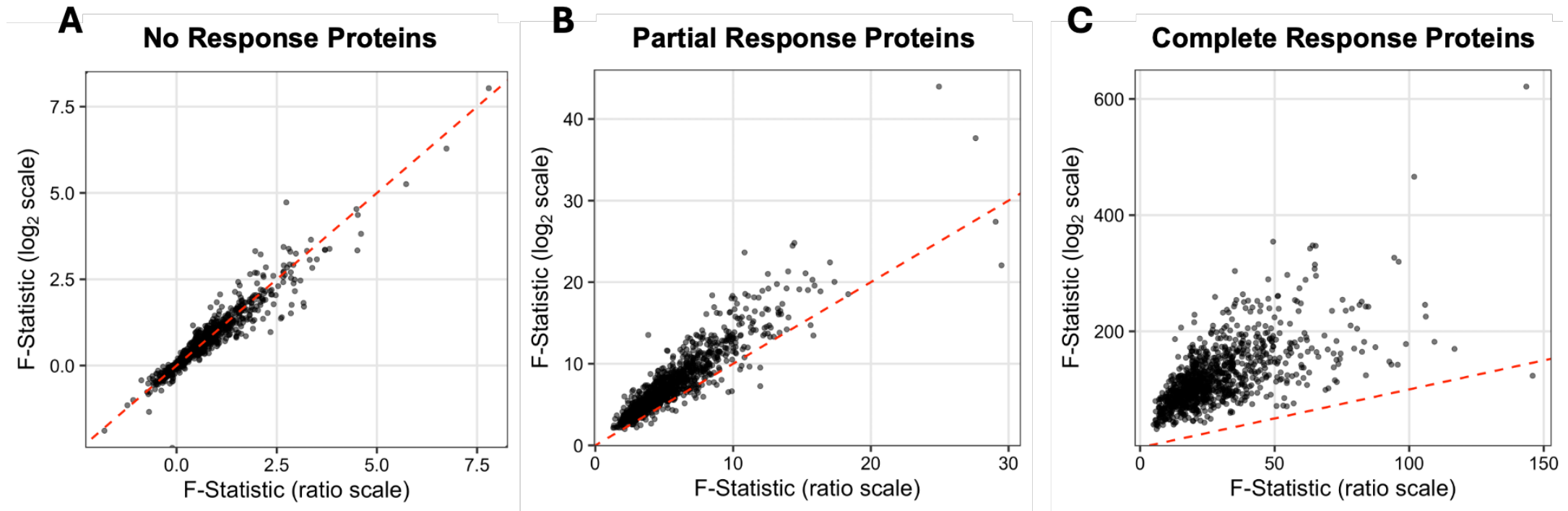

Figure 7: **Simulated dataset: target identification with MSstatsResponse on the log scale increased detection sensitivity without increasing false positives.** Simulation datasets containing 3,000 proteins (n = 1,000 in each strength group) were used to compare detection sensitivity on the log scale versus the ratio scale. **(A)** F-statistics for negative controls were similar on both scales. **(B)** Proteins with partial responses showed slightly larger F-statistics on the log scale compared to the ratio scale. **(C)** Similar patterns were observed for proteins with complete responses, although all F-statistics were sufficiently large to identify significance on both scales.

Task 3: OC50 estimation

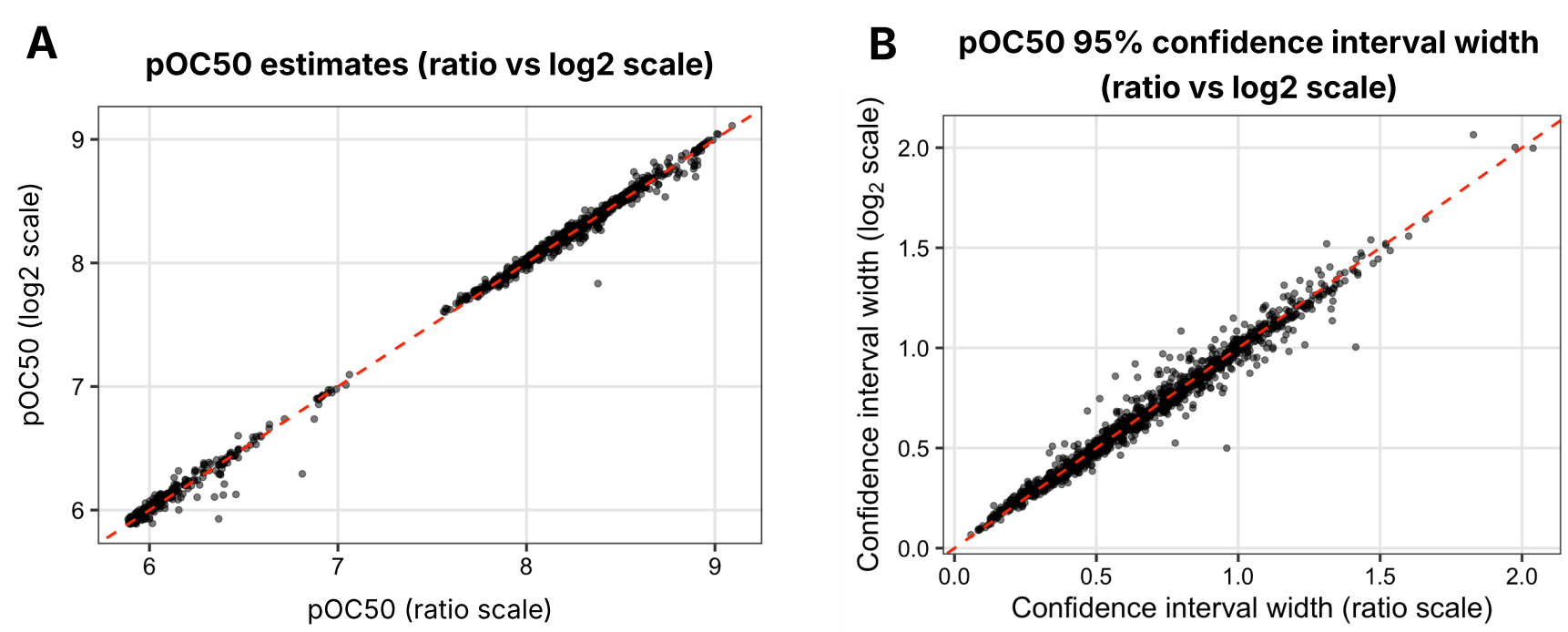

Figure 8: **Simulated dataset: OC50 estimates from MSstatsResponse are near identical on both the log and ratio scale.** OC50 values are reported as pOC50 ($\log_{10}$OC50), the default output of MSstatsResponse. Simulation datasets containing 3,000 proteins (n = 1,000 in each strength group) were used to compare pOC50 estimates and confidence interval widths on both scales. **(A)** pOC50 estimates follow a 1:1 trend regardless of what scale the data used to fit the model was. **(B)** shows that a similar trend was observed in 95% confidence interval widths.

4 Evaluation

4.1 Evaluation strategy

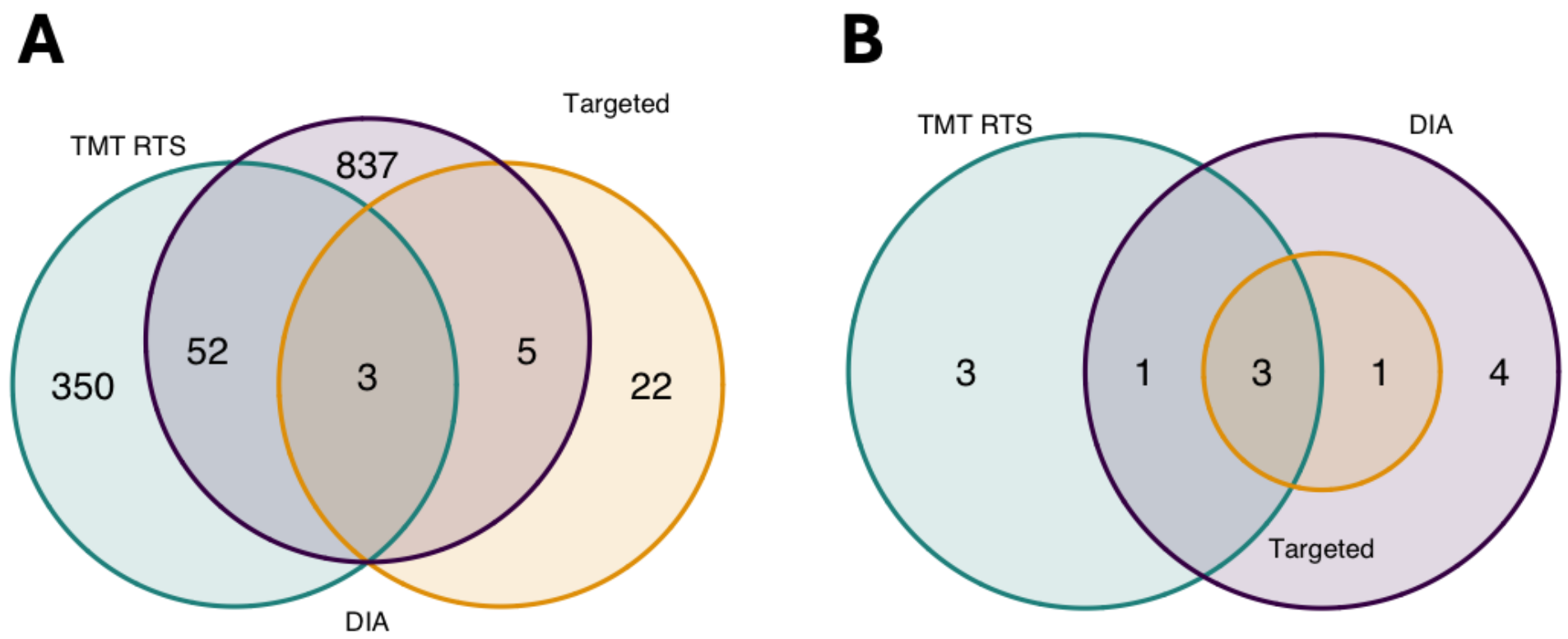

Figure 9: **DIA benchmark: dr4pl results were sensitive to how drug–protein interactions were defined.** (**A**) Using the OC50 estimate alone (i.e., requiring only that the OC50 95% confidence interval fall within the experimental dose range) produced a high number of false positives and substantial variability in the number of hits called across acquisitions. (**B**) A combined criterion that required both an in-range OC50 95% CI and a decreasing slope whose 95% CI excludes 0, produced more stable and reliable detection results. Three positive controls (SRC, CSK, LCK) were consistently detected across all acquisitions, while GAK was shared between TMT and DIA, and YES was shared between Targeted and DIA.

4.2 All acquisition strategies were suitable for chemoproteomics experiments

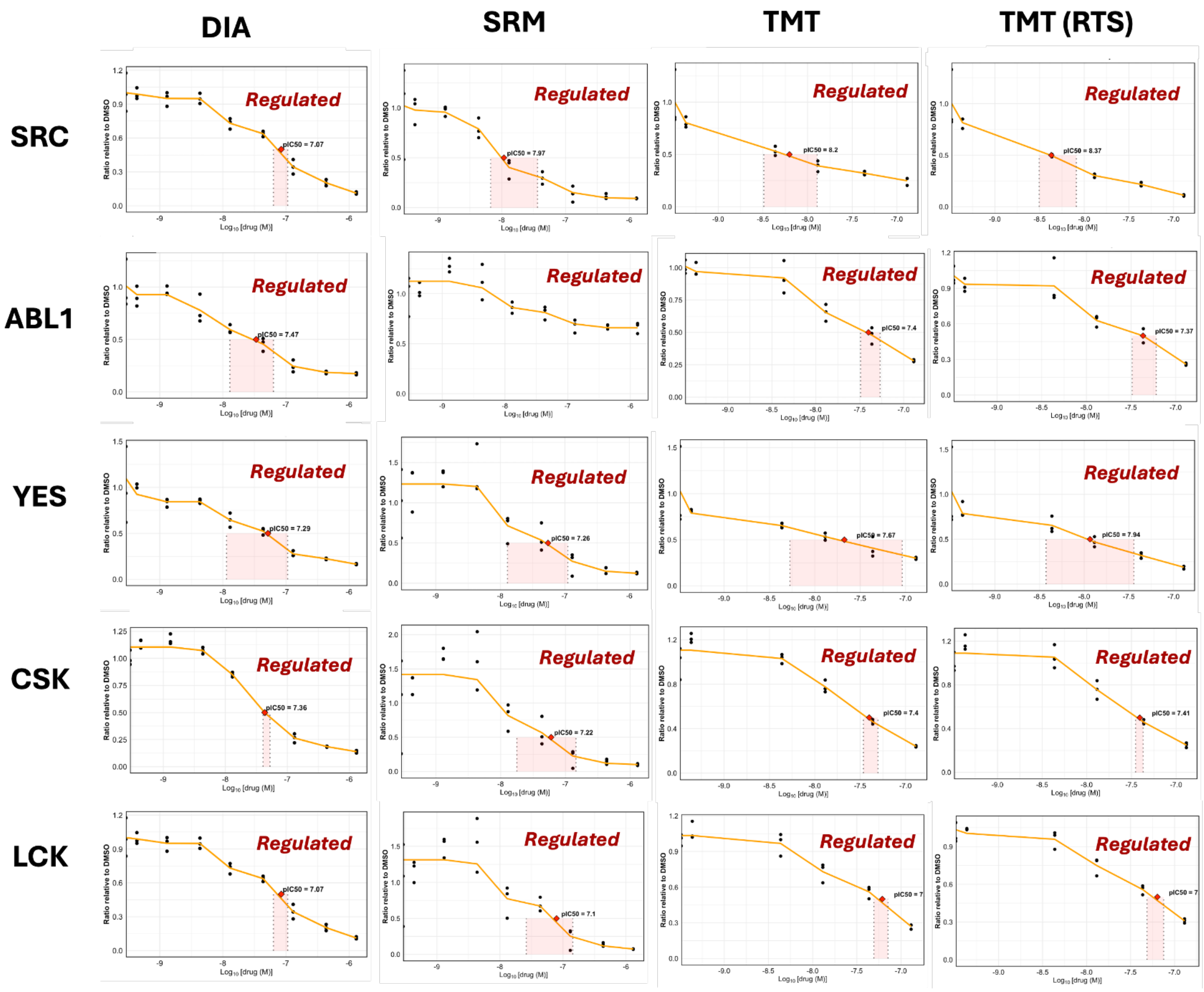

Figure 10: **Benchmark dataset: all positive controls exhibited a decreasing response upon increasing the dose of dasatinib.** Similar patterns were observed in all four benchmark datasets. In cases where 50% inhibition is not met (e.g., ABL1 in SRM benchmark), MSstatsResponse does not return OC50 estimates. In the TMT benchmark, YES was a borderline case with an adjusted p-value of 0.06 (cutoff = 0.05).

4.3 In experimental datasets, statistical methods produced distinct dose-response curves

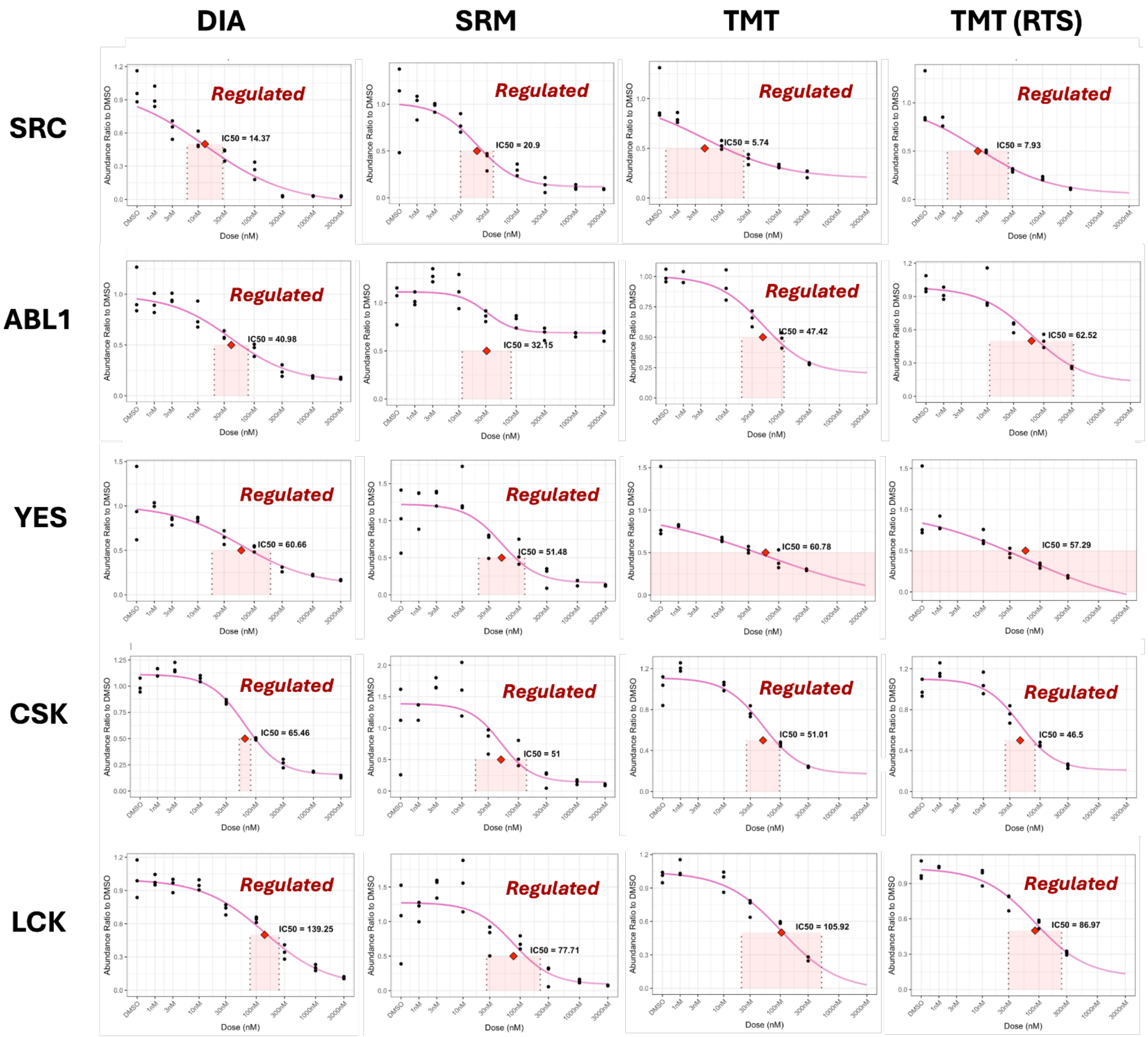

Figure 11: **Benchmark dataset: dr4pl response curves of all positive control proteins across acquisition benchmark datasets.** Similar patterns were observed in all four benchmark datasets. In cases where a sigmoidal trend was not observed (e.g., YES in the TMT and TMT-RTS benchmarks), dr4pl was unable to accurately estimate OC50 values, resulting in unbounded or large confidence intervals.

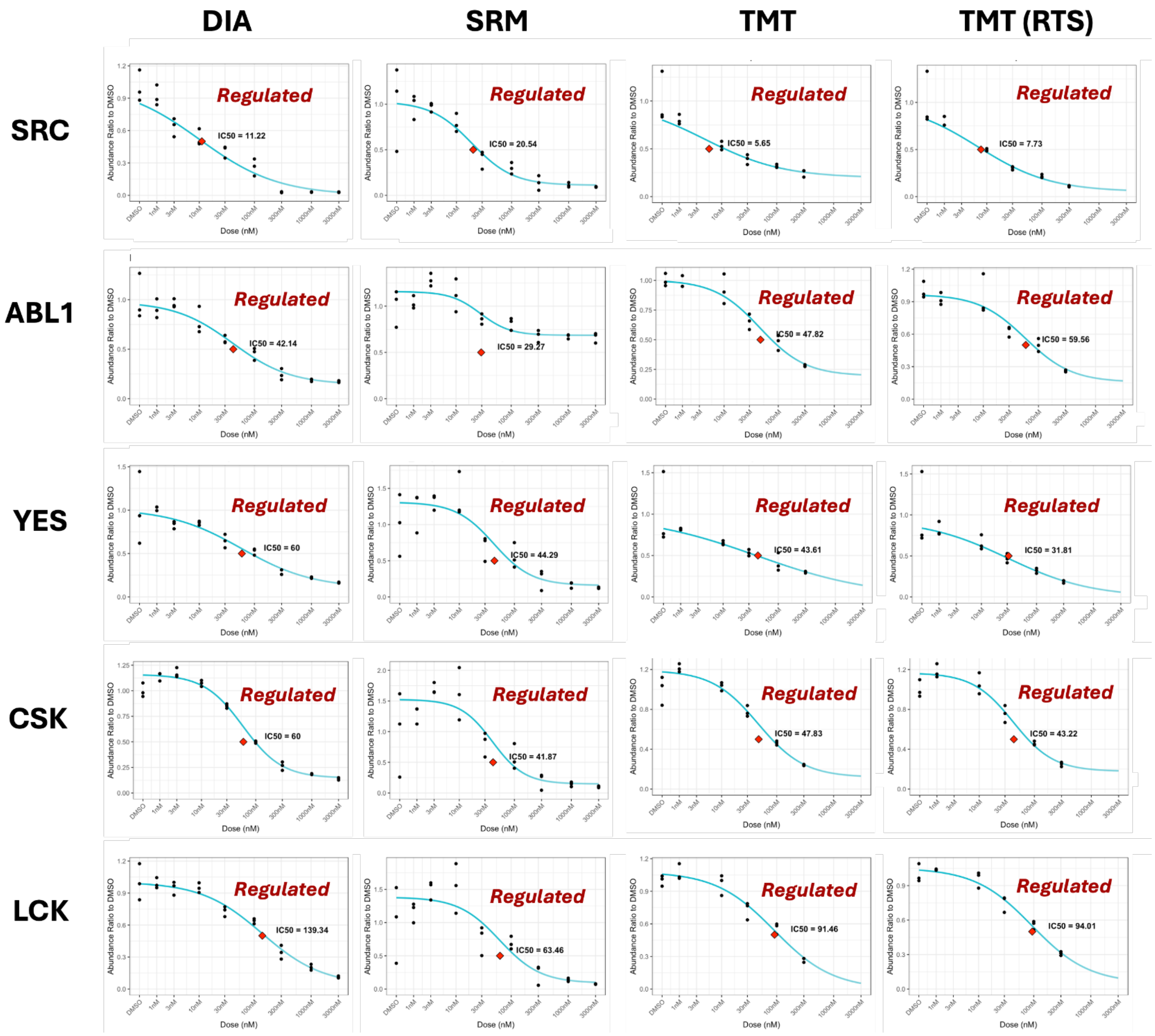

Figure 12: **Benchmark dataset: CurveCurator response curves of all positive control proteins across acquisition benchmark datasets.** Similar patterns were observed in all four benchmark datasets. In cases where partial inhibition is observed (e.g., ABL1 in SRM benchmark), CurveCurator failed to pick up the interaction.

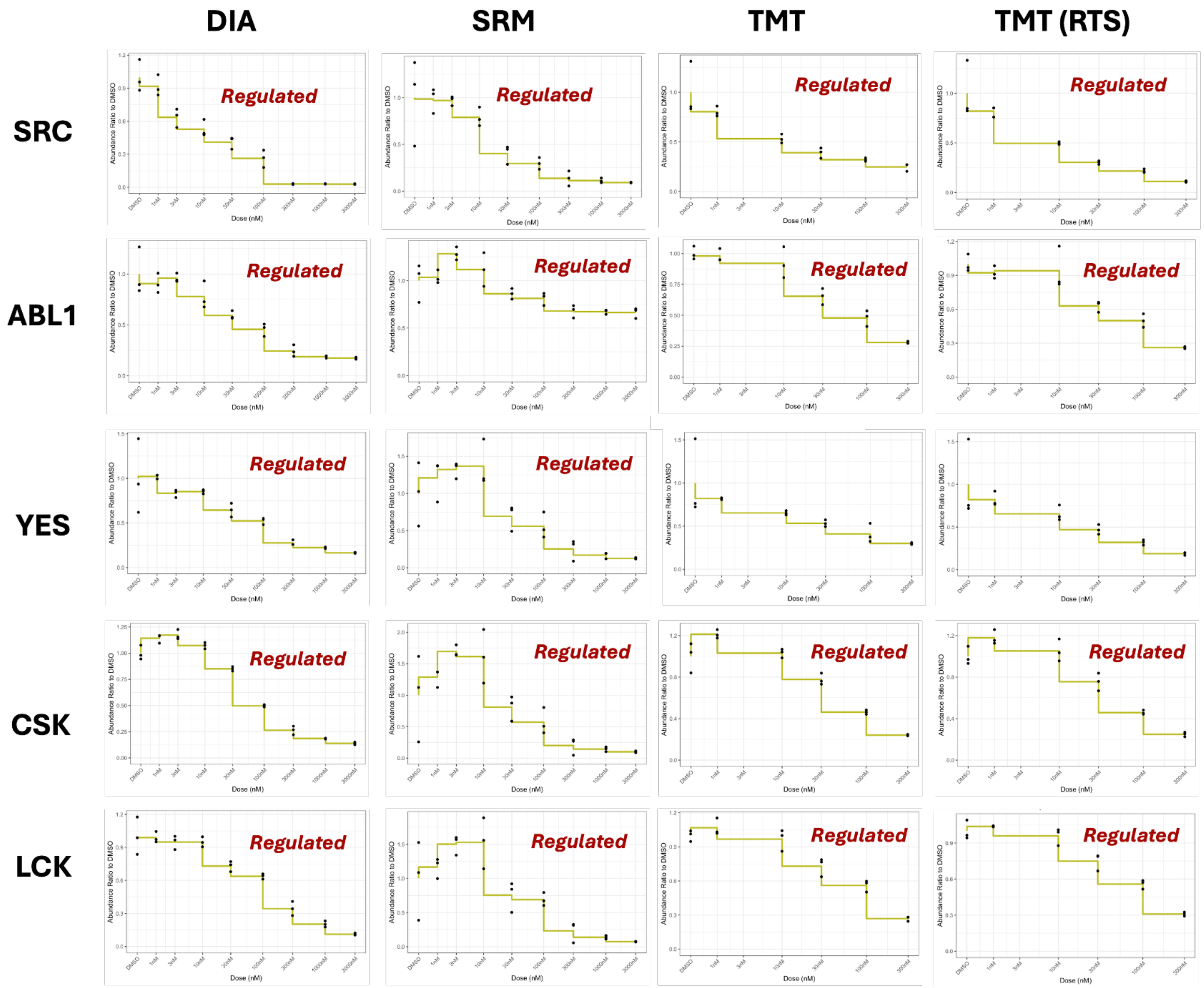

Figure 13: **Benchmark dataset: ANOVA response curves of all positive control proteins across acquisition benchmark datasets.** The ANOVA model fits to group means exactly, and is not well suited for does-response trend visualizations. Response patterns varied across proteins. For example, LCK and CSK in the SRM benchmark showed an initial increase followed by a decrease in abundance. ANOVA also failed to detect YES protein in both TMT benchmarks.

4.4 In experimental datasets, statistical methods differed in drug-target detection sensitivity

	TMT doses [0, 1, 10, 30, 100, 300]				All doses	
Method	DIA	TMT	TMT w/RTS	SRM	DIA	SRM
drc	0	5 (2)	5 (1)	0	5 (4)	2 (0)
dr4pl	2 (2)	6 (3)	7 (3)	0	9 (5)	4 (4)
CurveCurator	13 (5)	10 (5)	12 (5)	6 (4)	31 (5)	9 (4)
ANOVA	18 (5)	21 (5)	22 (5)	1 (1)	90 (5)	5 (5)
MSstatsResponse	4 (4)	9 (4)	11 (5)	1 (1)	14 (5)	5 (5)

Table 2: **Benchmark dataset significant drug-protein interactions across acquisition types and methods.** Columns 1–4 report results using six drug doses (DMSO, 1nM, 10nM, 30nM, 100nM, 300 nM) across DIA, TMT, TMT with RTS, and Targeted datasets. Columns 5–6 expand to include all available doses for DIA and Targeted (including 3 nM, 1 µM, and 3 µM). The number of significant proteins is shown for each method. Known Dasatinib targets (SRC, ABL1, YES, CSK, and LCK) are shown in parentheses and serve as positive controls.

4.5 In pseudo-single-replicate experimental datasets, drug-target detection varied between replicates

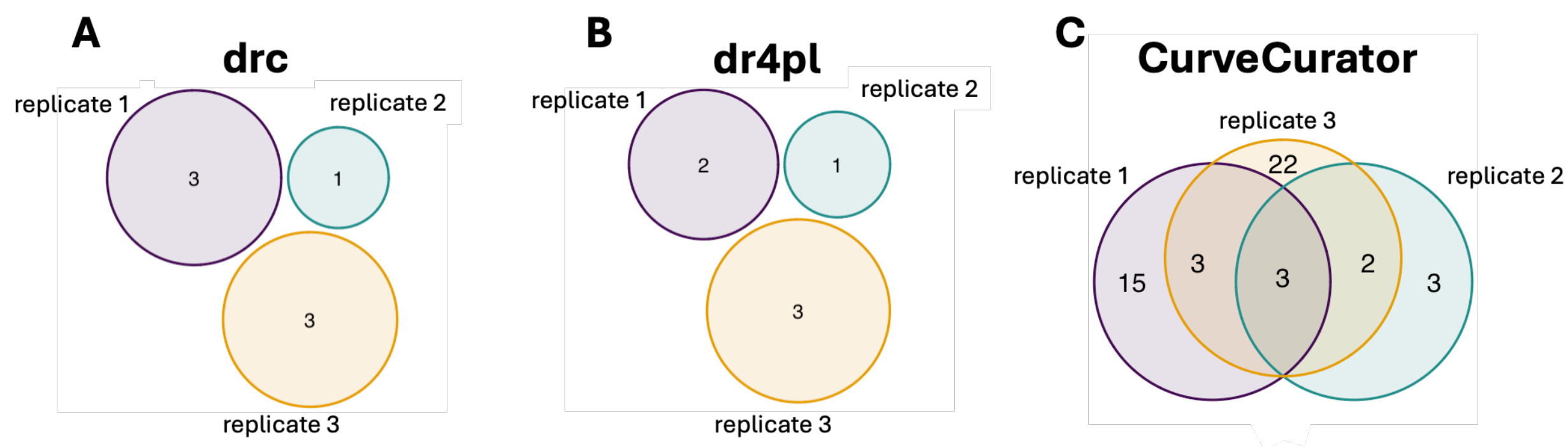

Figure 14: **TMT benchmark: single replicate analysis results vary greatly based on replicate choice.**

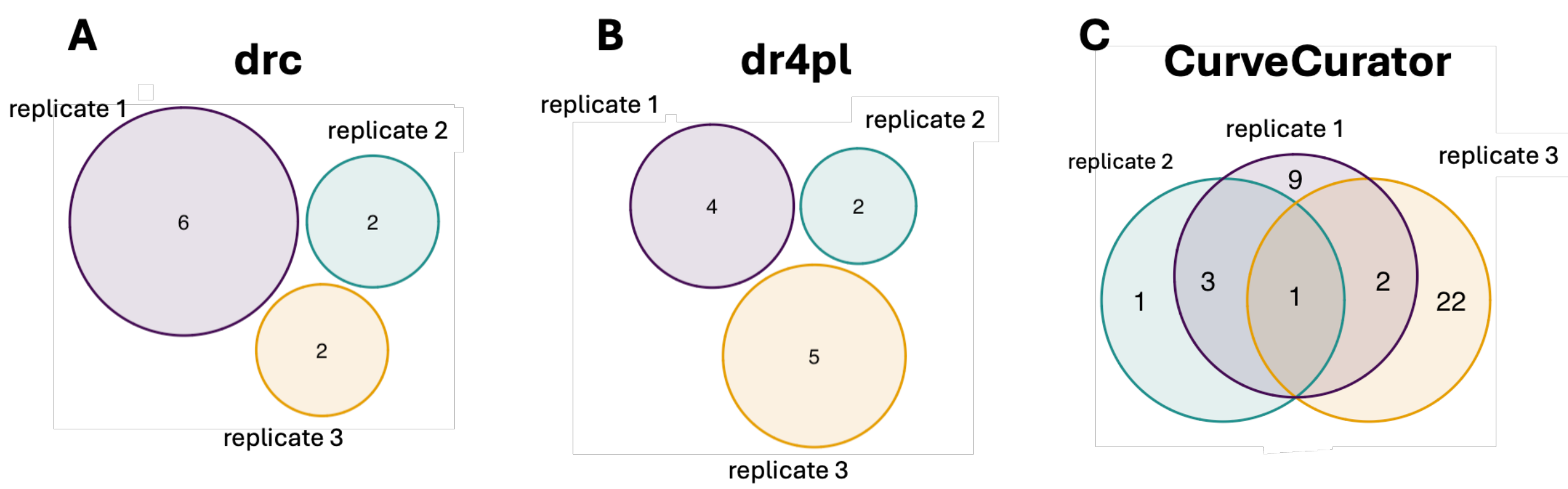

Figure 15: **TMT RTS benchmark: single replicate analysis results vary greatly based on replicate choice.**

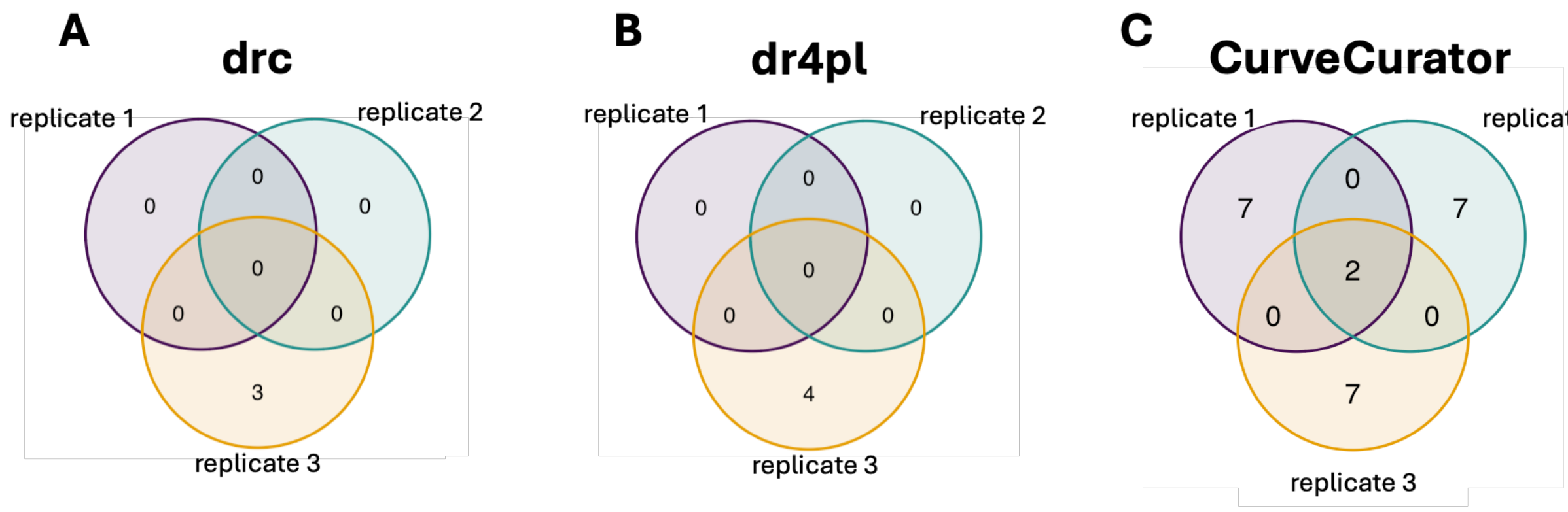

Figure 16: **SRM benchmark: single replicate analysis results vary greatly based on replicate choice.**

4.5.1 CurveCurator MAD-based quality control analysis

For the CurveCurator analyses, we performed additional analyses that followed the routine processing steps recommended by the developers, including the median absolute deviation (MAD) quality-control analysis. Following the documented guidance, samples with MAD < 0.10 are considered high quality, and samples above a MAD of 0.15 should be excluded [**Bayer2023**].

In the single-replicate analyses, each replicate was assessed independently. All per-replicate MAD scores (Supplementary Table 3) fell below the 0.15 exclusion threshold, so no samples were excluded.

When all three replicates were combined in the full benchmark, the DMSO MAD scores increased (Supplementary Table 4). In the full benchmark analysis, two out of the three DMSO replicates in the DIA dataset exceed the 0.15 exclusion threshold (0.3369 and 0.305). This variability was only detectable when all replicates were present, and is undetectable in the single replicate scenarios. To check whether it affected the conclusions, we repeated the full benchmark analysis two ways, using DMSO as the reference control (analysis used in the main) and using the lowest drug dose (1 nM) as the reference control. The two versions gave consistent results (Supplementary Figure 17), and the conclusions were unchanged.

We also repeated the single-replicate analysis using the lowest drug dose (1 nM) as the reference control in place of DMSO (Supplementary Figure 18). Single-replicate reproducibility remained poor under this alternative reference, indicating that the instability is a property of the single-replicate design rather than the quality of DMSO samples.

Table 3: **DIA and TMT benchmark, single-replicate CurveCurator analysis:** MAD scores for individual replicates. When assessed per replicate, all DMSO MAD scores fall below the 0.15 exclusion threshold and would not be removed by standard quality control.

Ratio	DIA Rep1	DIA Rep2	DIA Rep3	TMT Rep1	TMT Rep2	TMT Rep3
**DMSO**	**0.1095**	**0.0117**	**0.0693**	**0.0085**	**0.0353**	**0.0534**
1 nM	0.0565	0.0227	0.0466	0.0287	0.0241	0.036
10 nM	0.0767	0.0382	0.04	0.0338	0.0312	0.0378
30 nM	0.103	0.0454	0.0675	0.0459	0.0383	0.0441
100 nM	0.0549	0.0442	0.0697	0.0345	0.0344	0.0388
300 nM	0.0971	0.0661	0.177	0.0303	0.0196	0.0011
1000nM	0.0997	0.0369	0.097	–	–	–
3 nM	0.110	0.0314	0.0583	–	–	–
3000 nM	0.0654	0.039	0.0598	–	–	–

Table 4: **DIA and TMT full benchmark, all replicates CurveCurator analysis:** MAD scores for all replicates combined. When all replicates are combined, two of the three DIA DMSO samples exceed the 0.15 exclusion threshold (0.3369 and 0.305), and one TMT DMSO sample (0.1984).

Ratio	DIA	TMT
**DMSO 1**	**0.3369**	**0.1984**
**DMSO 2**	**0.305**	0.0587
**DMSO 3**	**0.111**	0.0601
1 nM	0.0639	0.0435
1 nM	0.0782	0.0522
1 nM	0.0531	0.0518
10 nM	0.0773	0.0447
10 nM	0.0472	0.0503
10 nM	0.0518	0.0514
30 nM	0.0942	0.0514
30 nM	0.0505	0.0587
30 nM	0.0872	0.054
100 nM	0.0693	0.0588
100 nM	0.0574	0.0559
100 nM	0.0632	0.0527
300 nM	0.121	0.0667
300 nM	0.0906	0.0759
300 nM	0.260	0.146
1000 nM	0.0843	–
1000 nM	0.0592	–
1000 nM	0.0894	–
3 nM	0.0772	–
3 nM	0.049	–
3 nM	0.0666	–
3000 nM	0.0904	–
3000 nM	0.0627	–
3000 nM	0.0783	–

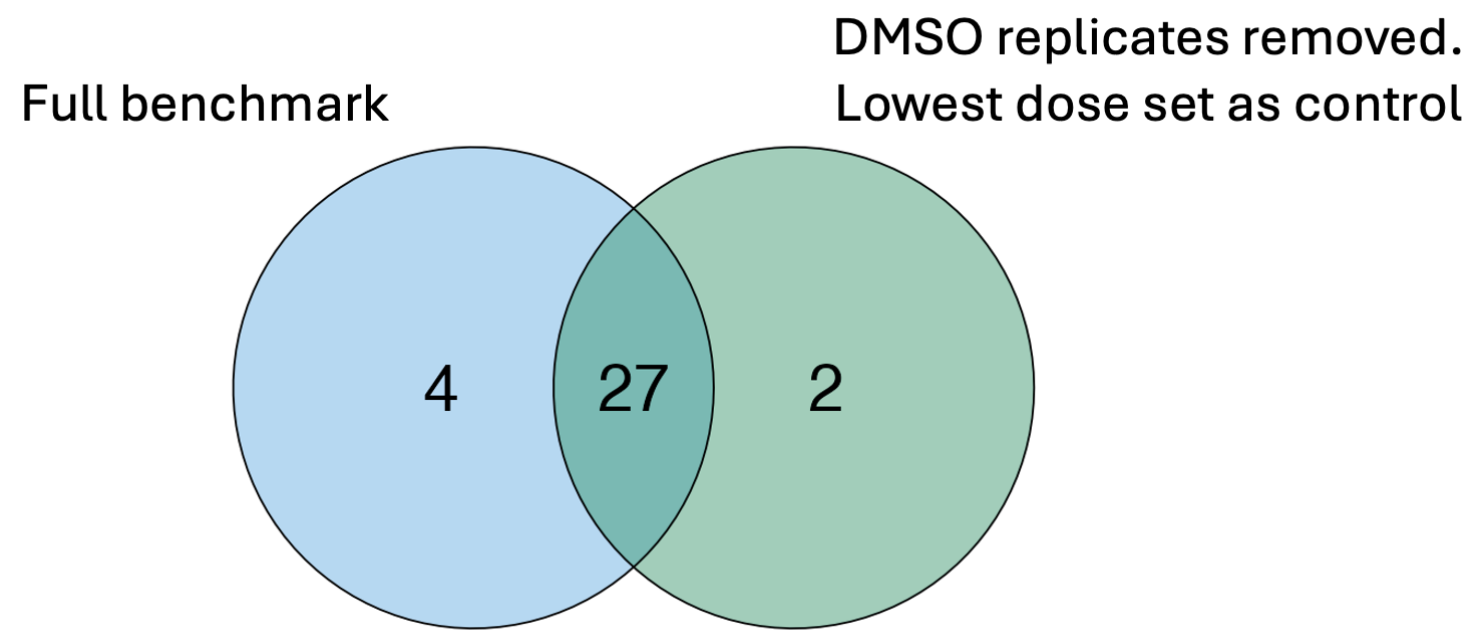

Figure 17: **DIA benchmark, all-replicate analysis by CurveCurator**: Replicated analyses using DMSO (full benchmark) or using 1nM drug dose as the control produced consistent results.

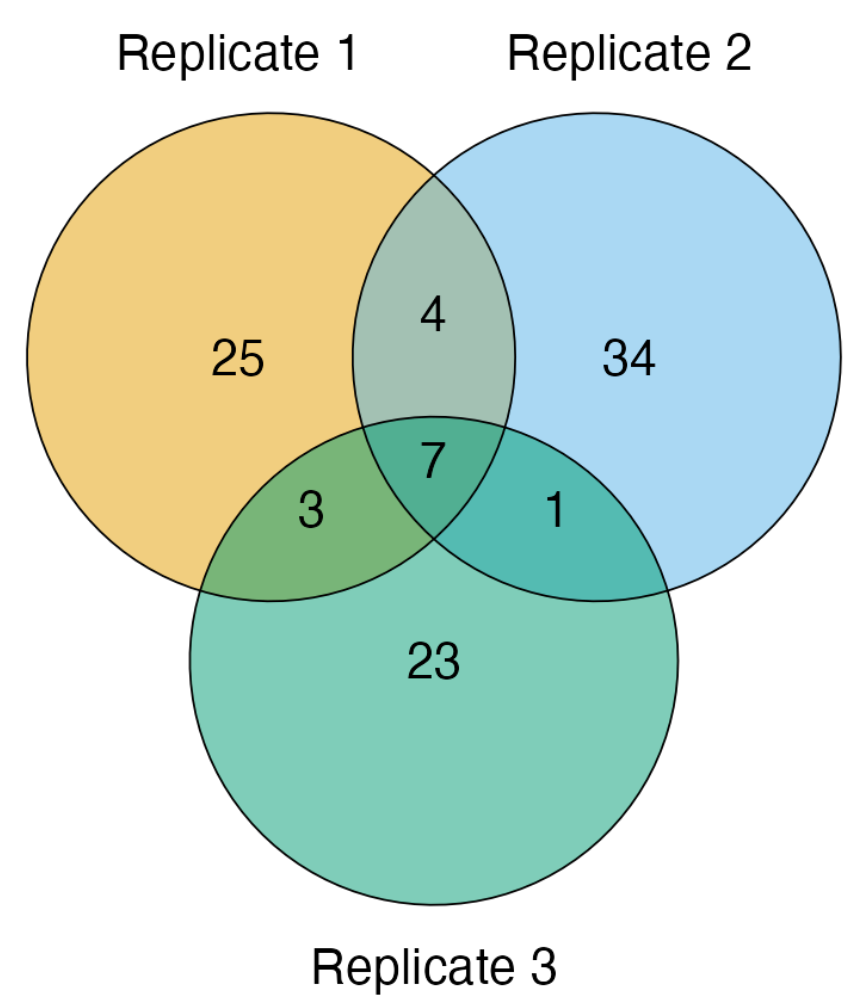

Figure 18: **DIA benchmark, lowest dose as control, single-replicate analysis by CurveCurator:** Single-replicate analyses had poor reproducibility after substituting DMSO with lowest drug dose.

4.6 In simulated datasets, MSstatsResponse detected partial responses without increasing false positives

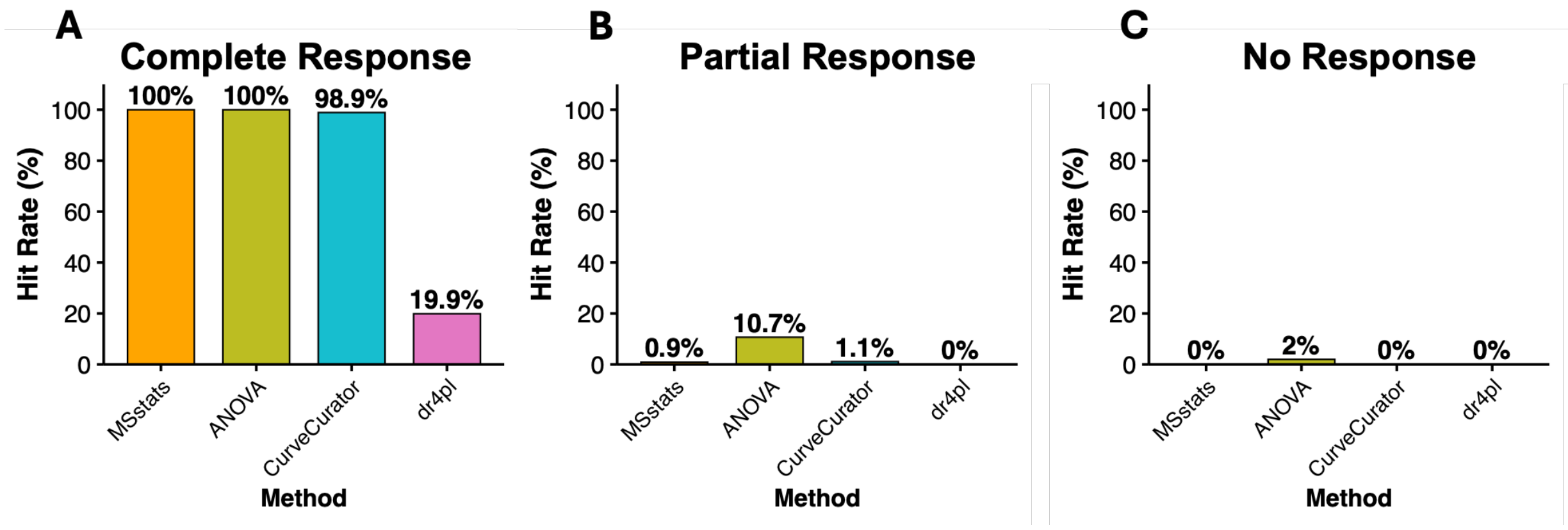

Figure 19: **Simulated dataset: method performance in high-variability confirmatory experiments.** Performance was evaluated on 3,000 simulated proteins across 9 doses (DMSO, 1nM, 3nM, 10nM, 30nM, 100nM, 300nM, 1000nM, 3000nM) with 3 replicates per dose and variance = 0.8. **(A)** Complete responses: dr4pl failed to handle high variability, with significantly decreased hit detection, while MSstats, ANOVA, and CurveCurator remained stable. **(B)** Partial responses: most methods failed to detect these, with only ANOVA picking up a small number of partial response. **(C)** Non-interactions: all methods correctly avoided false positives.

4.7 In simulated datasets with fewer doses, MSstatsResponse maintained drug-target detection sensitivity

		Acquisition method			
Dose count	Method	DIA	TMT	TMT-RTS	SRM
3 doses	drc	1 (0)	0	1 (0)	0
	dr4pl	0	0	0	0
	CurveCurator	24 (5)	10 (5)	11(5)	5 (4)
	ANOVA	12 (5)	8 (4)	16 (5)	5 (4)
	MSstatsResponse	6 (5)	6 (4)	10 (5)	3 (3)
1 dose	drc	0	0	0	0
	dr4pl	0	0	0	0
	CurveCurator	N/A	N/A	N/A	N/A
	ANOVA	6 (3)	3 (2)	2 (1)	0
	MSstatsResponse	2 (2)	3 (2)	2 (1)	0

Table 5: **Benchmark dataset significant drug–protein interactions across acquisition types and dose subsets, using 3 replicates.** Results are shown for analyses using a DMSO control and either a three-dose series (1 nM, 1 µM, and 3 µM for DIA and SRM; and 1 nM, 100 nM, and 300 nM for TMT) or a single high-dose treatment (3 µM for DIA and SRM, and 300 nM for TMT). Columns report the number of significant proteins detected for each acquisition method and analysis approach. Known Dasatinib targets (SRC, ABL1, YES, CSK, and LCK) are shown in parentheses and serve as positive controls.
